# Doppler-Resilient Link Adaptation for 3D Dual-Mobility Air-to-Vehicle Open RAN Networks

**DOI:** 10.3390/s26196144

**Published:** 2026-09-28

**Authors:** Adnan Alghammas, Ibrahim Elshafiey, Majid Altamimi

**Affiliations:** 1 Department of Electrical Engineering, King Saud University, Riyadh 12372, Saudi Arabia; ishafiey@ksu.edu.sa (I.E.); mtamimi@ksu.edu.sa (M.A.); 2 King Abdulaziz City for Science and Technology (KACST), Riyadh 12354, Saudi Arabia

**Keywords:** Open RAN, 3D vehicular networks, air-to-vehicle channel, Doppler spread, CSI aging, link adaptation, UAV-assisted V2X, Simu5G, smart cities

## Abstract

Low-altitude unmanned aerial vehicles (UAVs) serving as aerial base stations for ground vehicles create air-to-vehicle (A2V) links in which both endpoints move, compressing the channel coherence time so that the reported channel quality indicator (CQI) is already stale when applied. Link adaptation calibrated for terrestrial deployments does not account for this dual-mobility aging, and the resulting overestimation of link quality inflates first-transmission errors. This paper proposes a Doppler-aware CQI correction for three-dimensional (3D) A2V Open RAN networks: an offline-calibrated back-off, indexed by the maximum Doppler frequency and the Rician *K*-factor, is subtracted from the measured signal-to-interference-plus-noise ratio (SINR) before CQI quantization. Because the back-off depends only on parameters the network already derives from geometry and mobility, the correction acts from the first transmission and requires no feedback convergence, unlike outer-loop link adaptation (OLLA). The scheme is implemented in Simu5G within a 3D network model providing aerial cells, dual-mobility fading decorrelation, and an Open Radio Access Network (O-RAN)-based measurement plane, and evaluated across a factorial campaign spanning three schedulers, two deployment topologies, and paired random seeds. Applying the correction to A2V links alone reduces the first-transmission error rate of aerial-served vehicles by 46–54% in an urban grid with no penalty to terrestrial links; applied network-wide, it reduces total network error by 57–62% (urban) and 54–58% (highway) and mean latency by up to 0.38 ms, at a cost of 7.3–8.8 percentage points in resource-block utilization and negligible throughput loss. Against OLLA under identical conditions, it matches or improves the error rate in the urban grid with 2.7–3.8 percentage points less overhead and reduces highway error by a further 1.2–1.3 percentage points.

## 1. Introduction

Smart-city intelligent transportation systems (ITS) increasingly rely on unified Radio Access Network (RAN) architectures that can simultaneously serve ground vehicles, roadside units (RSUs), and low-altitude unmanned aerial vehicles (UAVs) [1,2,3]. In Next-Generation NodeB (gNB) deployments, the Open Radio Access Network (O-RAN) alliance architecture [4,5,6] disaggregates traditional RAN functions into vendor-neutral components managed by a multi-timescale control framework. Within this architecture, the Non-Real-Time (Non-RT) RAN Intelligent Controller (RIC) hosts long-horizon Non-RT RIC applications (rApps), whereas the Near-Real-Time (Near-RT) RIC executes latency-sensitive Near-RT RIC applications (xApps). The two control entities communicate via the A1 interface, while the Near-RT RIC observes and controls underlying E2 nodes over the E2 interface. For three-dimensional (3D) air–ground vehicular topologies, O-RAN provides a unified measurement and control plane that holistically manages aerial small cells, terrestrial gNBs, and mobile ground users.

This work investigates the wireless link established between a mobile UAV acting as an aerial small cell and an on-road vehicle, referred to herein as the Air-to-Vehicle (A2V) link. The A2V channel exhibits distinct propagation characteristics compared to conventional stationary-ground Air-to-Ground (A2G) links: both communication endpoints undergo independent, concurrent 3D motion. Consequently, the elevation angle and the corresponding Line-of-Sight (LoS) probability vary dynamically as both nodes navigate the environment. Furthermore, the overall Doppler spread depends on the 3D relative velocity vector rather than the ground vehicle speed alone. Neither the aerial user equipment (UE) model in 3rd Generation Partnership Project (3GPP) Technical Report (TR) 36.777 [7] nor the terrestrial vehicular frameworks in TR 38.901 and TR 37.885 [8,9] adequately captures this dual-mobility dynamic. Dual mobility severely degrades radio resource scheduling through accelerated Channel State Information (CSI) aging. In 5G New Radio (NR), link adaptation relies on channel quality indicator (CQI) reports generated by the receiver. By the time the Medium Access Control (MAC) scheduler processes a report, the feedback is already several milliseconds old. Because the modulation and coding scheme (MCS) selected from a CQI snapshot remains active until the next report arrives, rapid channel fluctuations render the snapshot stale. As a result, transport blocks are frequently transmitted using overly optimistic MCS selections, triggering excessive Hybrid Automatic Repeat Request (HARQ) retransmissions, increased latency, and wasted resource blocks (RBs). On A2V links, this mismatch occurs even at moderate ground speeds because the relative velocity depends on the motion of both the UAV and the vehicle and is exacerbated under Non-Line-of-Sight (NLoS) conditions where dominant specular paths are absent. From a control-plane perspective, an aged CQI stream represents a corrupted channel-state measurement, undermining the reliability of real-time camera, LiDAR, and telemetry data uploads.

To address this challenge, we treat the CQI feedback loop as an active measurement subsystem and evaluate the performance impact of a pre-quantization, channel-aware SINR correction. The experimental design meets two primary criteria: empirically measuring the isolated effect of the feedback correction and decoupling its performance gains from the underlying radio scheduler. The primary novelties of this work are threefold:

1. Integrated 3D Dual-Mobility A2V Model: A system-level channel framework implemented within Simu5G that computes small-scale fading decorrelation based on real-time 3D relative velocity vectors while supporting mobile aerial cells and handovers.
2. Doppler-Aware CQI Calibration: A scheduler-side CQI offset mechanism whose SINR back-off is systematically calibrated against the simulation engine’s link-adaptation curves as a function of maximum Doppler frequency and Rician *K*-factor, avoiding arbitrary heuristic tuning.
3. Reproducible O-RAN Measurement Framework: A multi-factorial, seed-paired experiment evaluating the proposed correction across three scheduling strategies, four feedback configurations, and two deployment topologies, benchmarked directly against standard Outer-Loop Link Adaptation (OLLA) using an O-RAN-based measurement plane.

The remainder of the paper is organized as follows. Section 2 reviews the related work and identifies the research gap. Section 3 presents the system-level architecture used to evaluate the CQI correction. Section 4 presents the dual-mobility channel model and the Doppler-aware CQI correction, including its calibration and the OLLA comparison arm. Section 5 describes the implementation and reproducibility of the OMNeT++/Simu5G framework. Section 6 defines the simulation scenarios, modeling assumptions, factorial design, statistical analysis, and simulation parameters. Section 7 presents the results, Section 8 discusses their implications and limitations, and Section 9 concludes the paper. Appendix A documents the measurement-plane implementation and key performance indicator (KPI) definitions, while Appendix B provides reproducibility and compatibility details.

## 2. Related Work

### 2.1. Air-to-Ground Channel Modeling for UAVs

The elevation-angle sigmoid for the LoS probability that this paper adopts was introduced by Al-Hourani, Kandeepan and Lardner [10]. It rises monotonically with the elevation angle, and its four parameter sets (suburban, urban, dense urban and high-rise urban) are still the reference for low-altitude platforms. Khawaja et al. [11] survey A2G measurement campaigns from ultra-high frequencies to millimeter waves and consolidate path-loss exponents, Rician K-factors and delay spreads. Matolak and Sun [12,13] report measured K-factors and shadowing statistics for over-water and near-urban settings, and are the source of the K-factor and shadowing parameters used here. TR 36.777 [7] consolidates the aerial-user study and, for its low-altitude cases, uses a LoS probability of the same sigmoid family. These models primarily consider a fixed or slowly moving ground node, whereas the A2V setting considered here requires the independent mobility of both the aerial and vehicular endpoints to be captured explicitly.

### 2.2. Air-to-Vehicle Links and Dual Mobility

Bian et al. [14] propose a 3D non-stationary wideband UAV-to-ground channel that captures the time variation caused by trajectory curvature and altitude changes; the ground node is quasi-static. Bai et al. [15] extend this to arbitrary 3D trajectories with UAV self-rotation and derive time-variant correlation functions and Doppler spectra, again with a static ground node. Kavas-Torris et al. [16] report measurements between connected automated vehicles and UAVs and observe that single-platform models under-estimate the Doppler spread of the joint motion. Zeng et al. [2] and Mozaffari et al. [1] both list the UAV-vehicle link among the open channel-modeling problems.

Several smart-city deployment scenarios motivate the need for a dual-mobility A2V model. Three come up repeatedly in the smart-city literature and are the ones we have in mind. First, UAV relays that follow platoons or convoys on highways, where the aerial cell moves with the traffic and vehicles overtake it in both directions [1,2]. Second, aerial edge servers that hover above intersections to receive perception streams from vehicles for cooperative sensing, in which the UAV re-positions to keep LoS while the vehicles pass under it [16]. Third, incident-response and traffic-monitoring UAVs that patrol a corridor and serve as temporary access points to the vehicles below. Across these scenarios, the independent motion of the aerial and vehicular nodes can produce substantial relative velocities and short channel coherence times, making timely CQI reporting and link adaptation important for reliable scheduling.

### 2.3. Terrestrial Vehicular Channels and CSI Aging

TR 38.901 [8] provides 3D channel models for NR, including the urban-macro (UMa) and rural-macro (RMa) path-loss, LoS-probability, and *K*-factor models used by the vehicle-to-infrastructure (V2I) component of the framework. TR 37.885 [9] defines the sidelink evaluation methodology, including the highway and urban vehicle-to-vehicle (V2V) path-loss models. Bazzi et al. [17] compare IEEE 802.11p and Long-Term Evolution vehicle-to-everything (LTE-V2X)and demonstrate the degradation of communication reliability with increasing vehicle density and link distance, highlighting the importance of capturing channel variability in vehicular-network evaluations. Clarke’s model [18] relates the Doppler spread to the coherence time and is the basis of the fading process used here. The consequence of a short coherence time for closed-loop link adaptation, namely that a reported CQI ages before it is used, is well known. Pedersen et al. [19] quantified the performance loss caused by delayed and noisy CQI in orthogonal frequency-division multiple access (OFDMA) scheduling, while Truong and Heath [20] analyzed channel aging in the context of multi-antenna systems. A common approach to mitigating link-adaptation mismatch is outer-loop link adaptation, which adjusts an SINR offset based on acknowledgment (ACK) and negative-acknowledgment (NACK) statistics [19,21]. However, a gap remains in jointly modeling A2V links with independently moving endpoints and in isolating, through controlled system-level experiments, the effect of a channel-aware CQI correction. To the best of our knowledge, prior work has not compared such a correction directly with OLLA under identical traffic, channel, and scheduler conditions.

### 2.4. O-RAN and Vehicular Control

Abdalla et al. [6] identify the Near-RT loop (10 ms to 1 s) as the timescale of radio resource management, and Polese et al. [5] describe the E2 service models through which key performance indicator (KPI) reports and control messages flow between xApps and E2 nodes. Moro et al. [22] apply xApp-based conditional handover to V2X services and report interruption-time reductions of 35–40%. Sroka and Kliks [23] use the RIC to steer vehicular traffic between cells and report reductions in macro-cell load of up to 30%. These works primarily consider the channel through reported link-quality information rather than explicitly modeling the underlying A2V channel observables. In contrast, our framework exposes the A2V channel observables to the RIC measurement plane and evaluates the scheduler-level effect of a physically motivated correction to the CQI feedback.

### 2.5. UAV-Assisted Vehicular Networks and Positioning

Mozaffari et al. [1] and Zeng et al. [2] provide comprehensive treatments of UAV-enabled cellular networks, highlighting altitude and 3D trajectory as important design variables affecting coverage and interference. Tataria et al. [3] identify UAV-aided access as a potential component of future 6G networks. Mohamed et al. [24] exploit delay-Doppler channel information in multi-UAV-assisted vehicular communications for joint millimeter-wave beamforming and UAV selection, illustrating the role of Doppler-domain information in mobility-aware aerial–vehicular connectivity. The system model adopted in this work follows this architectural perspective: UAVs operate as aerial cells within the same network as the terrestrial gNBs, and vehicles may transition between aerial and terrestrial serving cells. Taken together, the works reviewed above indicate a specific gap relevant to the present work. The A2G literature provides established LoS-probability and *K*-factor models, but the representative models considered here generally assume a fixed or slowly moving ground node. The dual-mobility studies characterize channel statistics but do not integrate them into a system-level simulation framework. Likewise, the O-RAN vehicular studies operate on reported link-quality information without explicitly modeling the underlying A2V channel. To the best of our knowledge, prior work has not quantitatively isolated, through controlled system-level experiments, the scheduler-level effect of a channel-aware CQI correction under A2V dual mobility, nor directly compared such a correction with outer-loop link adaptation under identical traffic, channel, and scheduler conditions. Table 1 summarizes the closest works with respect to these distinguishing characteristics. To address this gap, the proposed framework integrates within a reproducible system-level simulator an A2V channel with dual-mobility Doppler and an elevation-dependent *K*-factor, a CQI feedback correction calibrated against the simulator’s own link adaptation, and the experimental design that isolates the correction from scheduler effects and enables a direct comparison with OLLA.

## 3. System-Level Architecture for CQI-Correction Evaluation

This section defines the system-level architecture used to evaluate the proposed CQI correction under joint aerial and vehicular mobility. The architecture combines aerial and terrestrial serving cells, mobile vehicular UEs, and an O-RAN measurement plane. This arrangement allows the proposed feedback correction to be evaluated on dual-mobility A2V links while terrestrial V2I links provide an internal reference under matched traffic, scheduler, and network conditions. Figure 1 summarizes the architecture, while Section 5 identifies its implementation in OMNeT++/Simu5G.

### 3.1. Tiers and Nodes

The network covers a service area $A\subset R^{2}$ and has three tiers. The three-tier topology places the target dual-mobility A2V links and conventional terrestrial V2I links within the same system-level experiment, allowing the effect of the proposed correction to be separated by serving-cell class rather than inferred from independent simulations. The aerial tier comprises a set M={1,…,M} of rotary-wing UAVs, each carrying a complete NR gNB protocol stack. Rotary-wing platforms are assumed throughout because the patrol patterns defined in Section 6, including closed circular trajectories and reversing road-aligned legs at 7.5–15 m/s, as well as the hover-based energy model, require this platform type. These nodes are referred to as aerial distributed units (DUs) to distinguish their role as serving cells from that of user equipment (UE). UAV *m* has position $q_{m}\left(t\right)={\left[x_{m},y_{m},h_{m}\right]}^{⊤}$ and 3D velocity $v_{m}\left(t\right)$, patrols at a cruise altitude of 100 m (urban) or 120 m (highway) with a small-cell transmit power of 30 dBm, and is connected to the core over an idealized wireless backhaul (Section 6). The terrestrial tier comprises a set G={1,…,G} of fixed gNBs with antenna heights of 25 m in the urban scenario and 30 m in the highway scenario, each transmitting at 46 dBm. The vehicular tier is represented by the set K(t) of vehicles present at time *t*, whose mobility is generated by the SUMO road-traffic simulator; vehicle *k* has position $p_{k}\left(t\right)={\left[x_{k},y_{k},1.5\right]}^{⊤}$ and horizontal velocity $v_{k}\left(t\right)$. Each vehicle operates as an NR user equipment (UE) and associates with the strongest available aerial or terrestrial cell, performing handover when a neighboring cell becomes stronger according to the configured association criterion. All vehicles in the reported runs belong to the enhanced mobile broadband (eMBB) slice and generate constant-bit-rate (CBR) uplink traffic. Only the eMBB slice is exercised in the experiments reported here.

### 3.2. O-RAN Measurement Plane

The Non-RT RIC and the Near-RT RIC are OMNeT++ modules connected as in the O-RAN reference architecture, but in this study they act as a measurement plane. The Near-RT RIC subscribes, at the network module, to the MAC and physical-layer (PHY) signals emitted by every UE and cell: the HARQ first-transmission error rate, the MAC delay, the MAC throughput, the physical resource blocks (PRBs) served per transmission time interval (TTI) and the serving cell. It also subscribes to the observables emitted by the A2V channel modules: maximum Doppler, LoS Doppler shift, elevation angle, K-factor and the back-off applied. Every 10 ms it aggregates them into per-UE, per-cell and per-cell-class (aerial-served vs terrestrial-served) KPIs and records them. Four rule-based xApps (resource allocation, mobility management, slicing, energy management) consume these reports, and two rApps in the Non-RT RIC (slice policy, fleet management) exchange A1 messages with the Near-RT RIC every second. None of the xApp or rApp decisions are delivered to the Simu5G scheduler; the RIC therefore operates in shadow mode. This separation is deliberate: it prevents RIC control actions from confounding the measured effect of the CQI correction, allowing changes in HARQ reliability, delay, throughput, and PRB utilization to be attributed to the feedback condition and scheduler setting used in the factorial experiment. Accordingly, the paper makes no claim about closed-loop O-RAN control. The evaluated mechanism is therefore the Doppler-aware CQI correction at the radio node, while the O-RAN infrastructure provides synchronized observability of the channel conditions and resulting network-level response.

### 3.3. Interfaces and Scope Boundaries

The O-RAN interfaces are represented at the level required for the present measurement study. E2 indications are implemented through OMNeT++ signal subscriptions rather than encoded E2AP messages, and xApp-generated control messages are not applied to the scheduler. A1 exchanges between the Non-RT and Near-RT RICs are modeled with a 5 ms latency. Inter-cell signaling uses the Simu5G X2 mesh, with a 2 ms delay assigned to aerial inter-cell and backhaul links, while O1 configuration is represented through the simulation configuration files.

## 4. Dual-Mobility Channel Model and Doppler-Aware CQI Correction

### 4.1. Geometry and Notation

Table 2 lists the symbols used in this section and in Section 7; each is also defined where it first appears. For a link between an aerial node at q (velocity $v_{a}$) and a ground node at p (velocity $v_{g}$), let $d_{3D}=∥q-p∥$, let $d_{2D}$ be the horizontal distance, and let $\theta =\arctan\left(\left(h_{a}-h_{g}\right)/d_{2D}\right)$ be the elevation angle in degrees. The relative velocity is $v_{\mathrm{rel}}=v_{a}-v_{g}$; with $f_{c}=3.5$ GHz and *c* the speed of light, the maximum Doppler frequency of the link is

(1) $$f_{D}=\frac{f_{c}}{c}\;∥v_{\mathrm{rel}}∥,$$

and the Doppler shift of the LoS component is the radial projection

(2) $$f_{d}^{\mathrm{LoS}}=\frac{f_{c}}{c}\;v_{\mathrm{rel}}\cdot \hat{d},$$

where $\hat{d}$ is the unit vector from the aerial to the ground node.

Equations (1) and (2) retain the full three-dimensional relative motion between the aerial and ground nodes. The vertical component of the aerial-node velocity therefore contributes during ascent or descent, whereas a two-dimensional projection would omit it. For a terrestrial gNB, $v_{a}=0$ and both reduce to the vehicle speed and its radial component.

### 4.2. A2G Geometry: LoS Probability and Path Loss

The LoS probability follows the sigmoid of Al-Hourani et al. [10],

(3) $$P_{\mathrm{LoS}}\left(\theta \right)=\frac{1}{1+a\exp\;\left(-b(\theta -a)\right)},$$

with (a, b)=(9.61, 0.16) for the urban environment and (4.88, 0.43) for the suburban parameter set used on the open highway [10]. Figure 2a plots Equation (3) against altitude for three horizontal distances. The LoS state of a link is a Bernoulli draw with this probability, redrawn each time the relative displacement of the two nodes exceeds a correlation distance of 50 m. The path loss is the free-space loss at the 3D distance plus an excess loss η that depends on the LoS state,

(4) $$\mathrm{PL}=20\log_{10}d_{3D}+20\log_{10}f_{c}+32.44+\eta_{\mathrm{LoS}/\mathrm{NLoS}}\;\left[\mathrm{dB}\right],$$

with $d_{3D}$ in meters and $f_{c}$ in GHz. Here $\eta_{\mathrm{LoS}}$ and $\eta_{\mathrm{NLoS}}$ are the mean excess losses over free space in the LoS and NLoS states, that is, the additional attenuation caused by clutter and by diffraction around buildings; we use $\eta_{\mathrm{LoS}}=1.0$ dB and $\eta_{\mathrm{NLoS}}=20$ dB [10]. A zero-mean log-normal term is added for shadowing, with standard deviations $\sigma_{\mathrm{LoS}}=4.2$ dB in LoS and $\sigma_{\mathrm{NLoS}}=6.0$ dB in NLoS [12], correlated over the relative displacement of the two nodes with a 50 m decorrelation distance. Averaging Equation (4) over the LoS state gives the composite loss

(5) $$\bar{\mathrm{PL}}=P_{\mathrm{LoS}}\left(\theta \right)\;{\mathrm{PL}}_{\mathrm{LoS}}+\left(1-P_{\mathrm{LoS}}\left(\theta \right)\right)\;{\mathrm{PL}}_{\mathrm{NLoS}},$$

where ${\mathrm{PL}}_{\mathrm{LoS}}$ and ${\mathrm{PL}}_{\mathrm{NLoS}}$ are Equation (4) evaluated with $\eta_{\mathrm{LoS}}$ and $\eta_{\mathrm{NLoS}}$, and $P_{\mathrm{LoS}}\left(\theta \right)$ is Equation (3). The simulator does not use Equation (5) for a link. It draws the LoS state and applies the matching path loss of Equation (4). The composite serves only the comparison in Figure 2b, which sets the A2G path loss at 100 m altitude against the TR 38.901 UMa composite for a terrestrial gNB with a 25 m antenna. Both curves of Figure 2 are evaluated from the equations, not from simulation output. Below a horizontal distance of about 60 m the shorter terrestrial link has the lower loss. Beyond that distance the higher elevation angle raises the LoS probability, and the A2G loss is 8 to 14 dB lower. These large-scale components establish the instantaneous LoS state and path loss used by the A2V link; the proposed correction itself is driven by the mobility-dependent fading quantities introduced next.

### 4.3. Small-Scale Fading and Dual-Mobility Doppler

The small-scale fading of an air–ground link is Rician when a LoS path exists. The Rician K-factor *K* is the ratio of the power received through the dominant (specular) path to the power received through all diffuse (scattered) paths; K→0 recovers Rayleigh fading and large *K* approaches a non-fading channel. We call it geometric because, in the A2G measurements of Matolak and Sun [12,13], it appears to be governed by the scattering geometry rather than by the speed of either node. As the elevation angle grows, fewer ground scatterers fall into the first Fresnel zone of the direct path, the diffuse power drops, and *K* rises. We use the exponential fit

(6) $$K\left(\theta \right)=K_{1}\exp\left(K_{2}\;\theta \right),$$

Here, *K* in Equation (6) is expressed on a linear power-ratio scale and is converted to decibels when reported. With $K_{1}=2.0$ and $K_{2}=0.05$ $\deg^{-1}$, which gives 5.2 dB at $\theta =10^{\circ }$, 9.5 dB at $30^{\circ }$ and 13.9 dB at $50^{\circ }$, inside the 5 to 13 dB band that those campaigns report at comparable geometries and consistent with the sub-6 GHz surveys of Khawaja et al. [11]. Speed does not enter Equation (6); it enters through the Doppler shift and hence the temporal correlation of the fading.

The complex channel gain of the link is

(7) $$h\left(t\right)=\sqrt{\frac{K}{K+1}}\;e^{\;j(2\pi f_{d}^{\mathrm{LoS}}t+\varphi_{0})}+\sqrt{\frac{1}{K+1}}\;g\left(t\right),$$

where g(t) is a unit-power Clarke process realized as a sum of N=8 sinusoids whose Doppler frequencies are $f_{D}\cos\psi_{i}$ with angles of arrival $\psi_{i}$ drawn uniformly, and whose per-path delays give a mild frequency selectivity across the resource blocks (root-mean-square delay spread 50 ns, in line with the small A2G delay spreads of [12]). The autocorrelation of *g* is $J_{0}\left(2\pi f_{D}\tau \right)$ [18], so the coherence time of the diffuse part is set by $f_{D}$ of Equation (1), that is, by the joint relative velocity. The key distinction from a single-mobile-endpoint A2G model is that the Doppler spectrum and fading correlation are driven by the full joint 3D relative velocity $v_{\mathrm{rel}}$. Consequently, the resulting maximum Doppler frequency $f_{D}$, which determines the rate of CSI aging, also serves as one of the physical inputs to the proposed feedback correction in Section 4.5. In NLoS the LoS term is dropped (K=0). The oscillator phases advance step by step with the current $f_{D}$. The process therefore stays continuous when the relative speed changes, and between two calls it depends only on the elapsed time. This last property lets a CQI report computed at time *t* and a transport block received at t+τ see the same realization, aged by τ. The residual LoS Doppler shift also raises an inter-carrier-interference (ICI) floor $P_{\mathrm{ICI}}\approx {\left(\pi f_{d}^{\mathrm{LoS}}/\Delta f\right)}^{2}/3$ at the 30 kHz subcarrier spacing Δf. It is applied as $\mathrm{SINR}/(1+\mathrm{SINR}\;P_{\mathrm{ICI}})$ on both the feedback and the data path, without assuming any Doppler pre-compensation at the receiver. Section 7.6 reports how large it was in the runs. Figure 3 plots $f_{d}^{\mathrm{LoS}}$ and the coherence time $0.423/f_{D}$ against the vehicle speed for a UAV that moves against it; at the vehicle speeds of the highway scenario the coherence time is already inside the 1 to 7 ms window in which a CQI report is used.

### 4.4. Terrestrial Reference Links

The terrestrial V2I links provide a reference class against which the effect of dual-mobility A2V propagation and the proposed CQI correction can be resolved within the same system-level experiment. The V2I link between a vehicle and a terrestrial gNB uses the TR 38.901 UMa (urban) or RMa (highway) path loss, distance-dependent LoS probability, and shadowing as implemented in Simu5G, with the vehicle antenna at 1.5 m. Its small-scale fading uses the same process as Equation (7) with the vehicle speed in place of the relative speed and, for LoS links, the TR 38.901 mean K-factor of the scenario (9 dB UMa, 7 dB RMa); NLoS links are Rayleigh. For consistency across serving-cell classes, the terrestrial links use the same time-continuous fading formulation as the A2V links, with vehicle speed replacing the joint relative speed. This avoids introducing a different fading-update mechanism into the aerial–terrestrial comparison. Simu5G’s default Jakes process was therefore replaced because its speed estimate is obtained from finite differences of the Veins position updates and becomes zero between updates, which would otherwise make the terrestrial links artificially quasi-static. The framework also retains the TR 37.885 V2V sidelink models for the highway (two-slope, 80 m breakpoint) and urban (single-slope with 15 dB NLoS-vehicle blockage) cases. No sidelink traffic is generated in the experiments reported here; consequently, the V2V model does not contribute to the results analyzed below.

### 4.5. CSI Aging and Doppler-Aware CQI Correction

#### 4.5.1. Proposed CQI Correction

The proposed mechanism addresses CSI aging by converting two physically observable channel quantities—the maximum Doppler frequency $f_{D}$ and the Rician *K*-factor—into a pre-quantization SINR correction. Unlike a fixed margin, the correction adapts to both the rate of channel decorrelation and the strength of the specular component. Its magnitude is not selected heuristically; it is calibrated against the link adaptation used by the simulator so that the aging-induced excess first-transmission BLER is reduced toward the corresponding aging-free reference.

Simu5G computes a CQI report in three steps. The cell evaluates the SINR of the UE at the report instant, rounds it to an integer number of decibels, and selects the CQI class whose tabulated block error rate (BLER) at that SINR is closest to a $10^{-2}$ target. The report is refreshed every six TTIs with a one-TTI delay (Simu5G’s default fbPeriod and fbDelay), so a transport block is scheduled with a CQI that is between 1 and 7 ms old. When the channel decorrelates within that window, the realized first-transmission BLER exceeds the target, and the excess grows with $f_{D}$ and decreases with *K*.

The Doppler-aware correction acts at the point where the CQI is formed. Let ${\mathrm{SINR}}_{\mathrm{meas}}$ denote the SINR measured by the cell when the report is generated. The corrected value is

(8) $${\mathrm{SINR}}_{\mathrm{fb}}={\mathrm{SINR}}_{\mathrm{meas}}-\alpha \;\Delta \left(f_{D},K\right),\;{\mathrm{CQI}}_{\mathrm{eff}}=Q\left({\mathrm{SINR}}_{\mathrm{fb}}\right),$$

where Q(·) is the unchanged Simu5G quantizer described above and α is a scale factor, set to 1 in the main experiments and varied in the sensitivity study. The back-off therefore acts in the SINR domain before quantization, after which the unchanged quantizer maps the corrected SINR to the corresponding CQI index. Nothing on the data path changes; consequently, the underlying channel realization is identical with the correction enabled or disabled, enabling a controlled comparison.

The resulting correction is channel-state-dependent rather than fixed: $f_{D}$ captures how rapidly the CSI decorrelates, while *K* captures how strongly the channel is dominated by a persistent specular component. This two-variable dependence allows links with similar instantaneous SINR but different aging characteristics to receive different CQI corrections.

#### 4.5.2. Calibration of $\Delta (f_{D},K)$

We calibrated the back-off using a link-level study that reproduces the simulator’s link adaptation exactly (script aging_calibration.py). The study uses a flat Rician channel with the correlation of Equation (7), a report at *t* and a use at t+τ with τ uniformly distributed in [1, 7] ms, and an average SINR uniformly distributed in [0, 30] dB. It uses the typical-urban (TU) LTE-Sim BLER tables shipped with Simu5G, its integer-decibel quantizer with the $10^{-2}$ target, and its truncation rule at reception.

Each $\left(f_{D},K\right)$ point in the table is estimated from 120,000 independent report/use sample pairs. The candidate back-off is swept from 0 to 15 dB in 0.1 dB steps, and the entire calibration uses one fixed random seed so that the calibration script reproduces Table 3 exactly. For each $\left(f_{D},K\right)$ pair, we select the smallest Δ that brings the realized first-transmission BLER at the time of use back toward the aging-free reference, defined as the BLER obtained when report and use coincide.

For the NLoS row and for K≤3 dB, the aging-free reference cannot be reached at high $f_{D}$. In this regime, the aged SINR becomes nearly independent of the reported SINR, and the realized BLER approaches a floor that no finite back-off can remove. We therefore select the smallest Δ that recovers 90% of the attainable BLER reduction and enforce a non-decreasing Δ with respect to $f_{D}$. The 90% level is used as a practical saturation criterion: beyond it, the BLER-versus-Δ curve is nearly flat, so a larger threshold would increase both Δ and its associated resource cost for little additional reliability, whereas a substantially smaller threshold would under-correct the links that age most rapidly.

Table 3 gives the resulting calibration, and Figure 4 shows the calibrated curves, the BLER inflation they correct, and the sensitivity to the feedback window. Two characteristics of the calibration are particularly important. First, the back-off saturates once $f_{D}\tau$ exceeds approximately 0.4, corresponding to roughly 50–100 Hz for the default feedback window, because the channel is already substantially decorrelated between report and use. Second, the required back-off is strongly dependent on *K*: an NLoS (Rayleigh) link requires approximately 8 dB, a LoS link with K=9 dB requires approximately 3.5 dB, and a link with K≥15 dB requires less than 1 dB.

The closed-form approximation

(9) $$\Delta \left(f_{D},K\right)\approx \Delta_{\max}\left(K\right)\left(1-\exp\left(-f_{D}/f_{\mathrm{ref}}\left(K\right)\right)\right)$$

fits each row with a root-mean-square error below 0.3 dB ($\Delta_{\max}$ ranges from 7.9 dB as K→0 to 0.3 dB at 20 dB, while $f_{\mathrm{ref}}$ ranges from 27 to 78 Hz). The simulator nevertheless uses bilinear interpolation of the calibrated table rather than the analytical fit. Section 7.7 examines the effect of shortening or lengthening the feedback window.

#### 4.5.3. Outer-Loop Link Adaptation as the Comparison Arm

A standard remedy for link-adaptation mismatch is outer-loop link adaptation (OLLA) [21]. OLLA maintains one offset $\delta_{u}$ per UE and updates it from the decoding outcome of each first transmission. A negative acknowledgment increases the offset by $\Delta_{\mathrm{up}}$, while a positive acknowledgment decreases it by $\Delta_{\mathrm{down}}=\Delta_{\mathrm{up}}\;t/\left(1-t\right)$, so that the offset converges toward an operating point at which the first-transmission BLER approaches the target *t*.

We implemented OLLA in the same channel module and apply its offset at the same pre-quantization point as the proposed back-off:

$${\mathrm{SINR}}_{\mathrm{fb}}={\mathrm{SINR}}_{\mathrm{meas}}-\delta_{u}.$$

We use $\Delta_{\mathrm{up}}=1$ dB, a first-transmission target of t=0.1, which is commonly used in LTE and NR link-adaptation studies [19], and constrain $\delta_{u}$ to [−3,12] dB. The offset is initialized to zero for every UE and updated from the uplink decoding outcome at the serving cell. With one uplink packet every 10 ms per vehicle, the offset changes by 1 dB after a NACK and by approximately 0.1 dB after an ACK. It therefore reaches its operating region within a few seconds after attachment, which is short relative to the mean vehicle lifetime of 66–77 s (Section 6.4). A tighter variant with $t=10^{-2}$, matching the per-block target used by the CQI quantizer, is evaluated in the sensitivity analysis.

The two correction mechanisms therefore differ in the information that drives them. OLLA reacts to each UE’s ACK/NACK history without explicit knowledge of the underlying channel state, whereas the proposed back-off responds directly to the Doppler frequency and Rician *K*-factor of the current link and requires no error-history convergence. Section 7 compares the two mechanisms under identical traffic, channel, and scheduler conditions.

Two remarks are important for interpreting the proposed correction. First, the back-off is reliability-oriented. It restores the block error rate toward the operating point for which the underlying link adaptation was designed, allowing the scheduler to account for mobility through a lower modulation and coding scheme rather than through additional retransmissions. A throughput-optimal correction would generally be smaller; the sensitivity study examines this reliability–resource trade-off through α.

Second, the aging-free reference corresponds to the performance that the simulator would reach when report and use coincide, so the numerical calibration is tied to Simu5G’s link adaptation. Although the numerical values of $\Delta (f_{D},K)$ depend on the underlying CQI quantizer, BLER curves, and feedback timing, the calibration procedure itself is not Simu5G-specific and can be repeated for another link-adaptation implementation using the same report/use experiment. The OLLA arm is therefore evaluated as an alternative to the proposed back-off rather than combined with it, allowing the individual effects of the two mechanisms to be compared directly.

## 5. Implementation and Reproducibility

### 5.1. Software Stack and Component Roles

This section documents how the proposed channel and CQI-correction mechanisms are integrated into the system-level simulator and distinguishes the components introduced for this study from those reused unchanged. The purpose is reproducibility rather than to attribute methodological novelty to the underlying simulation stack. The framework runs on OMNeT++ 6.0.3 [25], Simu5G 1.2 [26], INET 4.5, Veins 5.2 [27] and SUMO 1.18 [28], all built in release mode. Simu5G supplies the NR protocol stack: the PHY and MAC layers, adaptive modulation and coding (AMC), the schedulers, HARQ, radio link control (RLC), the packet data convergence protocol (PDCP), handover, X2 signaling and the user plane. INET supplies the Internet Protocol (IP) layer and the wired links. Veins couples OMNeT++ to the SUMO vehicle traces. Table 4 lists every module shown in Figure 1 and Figure 5, states whether it was written for this work or reused unchanged, and whether it affects the reported results. Figure 5 follows one aerial cell through the feedback path, the data path and the measurement plane, and marks the point at which the effective CQI is formed.

### 5.2. Integration of the CQI Correction in Aerial Distributed Units

The AerialDU module provides the mobile-cell context required to evaluate the dual-mobility A2V channel without modifying Simu5G’s NR protocol stack. Because the standard Simu5G gNodeB uses a static mobility submodule, AerialDU retains the gNodeB structure including the NR network interface, tunneling user plane, and X2 applications while replacing the static mobility component with the parametric UAV3DMobility module. The Simu5G PHY reads the current node position from its mobility module at every transmission and reception, and the UE handover procedure uses the position carried in each broadcast. Consequently, aerial-cell mobility can be introduced without modifying the underlying PHY, MAC, or handover procedures.

Aerial DUs use A2VChannel as their channel model. Because Simu5G forms the UE CQI at the serving cell, the proposed back-off of Equation (8) is applied exactly once at the pre-quantization feedback stage and is never applied to the data-path SINR. In the OLLA comparison arm, the offset $\delta_{u}$ is inserted at the same point, ensuring that the two correction mechanisms are evaluated through the same CQI-processing path. The vehicle-side instance of A2VChannel is used only for cell selection and handover and therefore applies neither the proposed back-off nor the OLLA offset.

For cell selection, the vehicle-side channel instance uses the antenna gain of the peer cell being evaluated (3 dBi for an aerial DU and 18 dBi for a terrestrial gNB), ensuring that the received-power comparison reflects the correct serving-cell configuration. Terrestrial gNBs use the same channel-module type but exercise only its V2I branch. Further details of the measurement-plane implementation and KPI definitions are provided in Appendix A, while reproducibility and compatibility details are given in Appendix B.

## 6. Experimental Setup

### 6.1. Scenarios and Their Purpose

Two scenarios are used, and each has a distinct purpose. The urban scenario is a 1000×1000 m SUMO road grid with four terrestrial gNBs in a 2×2 pattern. Three aerial DUs fly circular patrols of 150 m radius around their stations at 100 m altitude and 7.5 m/s, and vehicles drive at 20 to 60 km/h. Its purpose is the moderate-mobility regime, in which the CQI window and the coherence time are comparable and the elevation angles are low, so that about a third of the A2V links, and nearly all V2I links, are NLoS. The highway scenario is a straight 3000 m six-lane road (three lanes per direction) with six roadside gNBs at 500 m spacing. Five aerial DUs fly 500 m legs back and forth above the road at 120 m altitude and 15 m/s, and vehicles drive at 60 to 140 km/h in both directions. Its purpose is the high-Doppler regime, with the suburban LoS parameters of the open environment and aerial links that are mostly LoS at high elevation. During a run every vehicle attaches on entry, uploads its constant-bit-rate stream, hands over between cells as it drives, and leaves the simulation at the end of its route. The aerial DUs patrol, the terrestrial gNBs are fixed, and the RIC measures. The per-scenario settings, and the number of vehicles that the runs actually contained as read from the recorded serving-cell and KPI signals, are listed in Section 6.4. Together, the two scenarios test the correction under distinct combinations of Doppler rate and LoS strength, the two channel properties that determine the calibrated back-off $\Delta (f_{D},K)$.

### 6.2. Modeling Scope and Methodological Abstractions

To maintain computational tractability while ensuring rigorous experimental isolation, Table 5 classifies system modeling factors as retained, simplified, or omitted. The guiding principle retains all physical and MAC-layer mechanisms directly impacting the trajectory from 3D relative mobility to CQI quantization and HARQ outcomes. We simplify factors that cause uniform shifts across all experimental conditions, and omit parameters that introduce secondary variables.

Two key abstractions require explicit justification:

- Inter-Cell Interference: Excluded (following standard Simu5G defaults) because uplink aerial interference represents a separate optimization problem. While inter-cell interference would increase baseline error rates, it does not impact the underlying SINR back-off correction mechanism.
- Downlink Traffic: Excluded to isolate uplink CQI feedback aging. Downlink CQI reports undergo identical channel measurement and quantization pipelines, experiencing equivalent aging dynamics under dual mobility.

### 6.3. Factorial Design and Statistical Analysis

The main experiment is a 3×4×2 factorial with five replications. The first factor is the uplink scheduler: proportional fair (PF), deficit round robin (DRR) or maximum carrier-to-interference (MAXCI). The second factor is the feedback setting: off, Doppler-aware back-off on the aerial-DU links only, back-off on all links, or OLLA on all links as the comparison arm of Section 4.5. The OLLA arm is applied to all links so that its network-level effect can be compared directly with the all-links Doppler-aware correction under the same scheduler, traffic trace, and channel realization. The third is the scenario. Each cell of the design is run for 300 s of simulated time after a 10 s warm-up with seed sets 0 to 4. The SUMO seed equals the seed set, so all treatments of a repetition share the same traffic trace, and differences within a repetition are paired. Everything else (topology, channel physics, traffic, RIC) is identical across cells. We report three kinds of statistics. The first is the mean with a 95% confidence interval from Student’s *t* distribution over the five seeds. The second is the paired difference (feedback setting minus off, same scheduler, same repetition) with its confidence interval and the *p*-value of a paired *t*-test. The third is a two-way analysis of variance (ANOVA) per scenario and KPI, with the scheduler, the feedback setting and their interaction as effects. All treatments of a repetition share the same seed and traffic trace. The paired differences exploit this common-seed structure and constitute the primary inferential evidence. The two-way ANOVA is reported as a complementary summary of the scheduler, feedback, and scheduler-by-feedback effects and does not include seed as a separate factor. The primary reliability endpoint of the experiment is the HARQ first-transmission error rate at the level of the MAC protocol data unit (PDU). It is the quantity that CSI aging degrades and that the calibrated back-off is designed to restore, and the statistical conclusions of Section 7 rest on it. The mean and 95th percentile of the uplink MAC delay, the share of PDUs delivered at the first transmission, the per-UE MAC throughput and the PRB utilization are supporting KPIs. These supporting KPIs characterize the latency and resource consequences of the correction and are interpreted as secondary outcomes rather than independent primary hypotheses. This keeps the interpretation focused and limits the number of comparisons. Each KPI is also reported for the aerial-served and terrestrial-served subsets. The sensitivity study (highway, PF, three seeds) varies the scale α∈{0, 0.5, 1, 1.5, 2}, the K-factor coefficient $K_{1}\in \left\{1,\;2,\;4\right\}$, the CQI period {2, 6, 10} TTIs and the vehicle-speed scale {0.5, 0.75, 1}.

### 6.4. Simulation Parameters

Table 6 lists the physical-layer and network parameters shared by both scenarios, Table 7 the channel parameters with their sources, and Table 8 the per-scenario configuration. Parameters adopted from standards, measurement studies, and Simu5G defaults are distinguished from those calibrated or selected for this study, including the CSI-aging back-off $\Delta (f_{D},K)$ and the OLLA comparison settings defined in Section 4.5. The uplink MAC PDU to which the HARQ error rate refers is the unit scheduled by Simu5G per TTI and per UE and may span several PRBs. Section 7 returns to this point when interpreting the absolute error rates.

## 7. Results

This section reports the results in seven parts. They are the realized scenario conditions, the factorial results with their confidence intervals, the paired effect of the feedback correction on the primary endpoint (the HARQ first-transmission error rate), the comparison with OLLA and the supporting delay and resource KPIs, the delay distributions, the recorded A2V observables, and the sensitivity analysis. The simulator records all performance and channel quantities. The confidence intervals, paired differences, significance tests and ANOVA statistics are then computed from those recordings. The analytical quantities defined in Section 4 are not evaluated again here.

### 7.1. Realized Scenario Conditions

Across the five seeds, each urban run contained 244 vehicles entering the grid, with a mean simultaneous population of 56 vehicles and a mean vehicle lifetime of 66 s. Each highway run contained 93 entering vehicles, with a mean simultaneous population of 25 and a mean lifetime of 77 s (Table 8). The number of entering vehicles was the same for every seed because the SUMO demand file fixes the total demand. The seed changes only the departure times and the channel realizations.

Across the evaluated configurations, the three aerial DUs in the urban grid served 22 to 23% of the active vehicles, while the five aerial DUs above the highway served approximately 45%, with no material variation across schedulers or feedback settings. These shares follow from the received-power cell selection. An aerial DU transmits at 30 dBm through a 3 dBi antenna, whereas a terrestrial gNB transmits at 46 dBm through an 18 dBi antenna. An aerial DU is therefore selected mainly by vehicles close to its flight path. Its serving share is higher on the highway because it flies directly above the road, while the nearest roadside gNB can be as far as 250 m from a vehicle.

The operating conditions of the served links are detailed in Section 7.6. The median elevation angle is $38.1^{\circ }$ in the urban grid and $52.0^{\circ }$ on the highway. The corresponding aerial-link LoS shares are 65% and 100%, while the median Rician K-factors of the LoS aerial links are 12.0 and 14.3 dB, respectively. The terrestrial links differ markedly between the two scenarios. In the urban grid, only approximately 9% of the V2I evaluations are LoS under the UMa model; the remaining 91% are NLoS, which the model treats as Rayleigh fading, and receive the largest calibrated back-off. By contrast, 90% of the highway V2I evaluations are LoS under RMa.

The two scenarios therefore cover different propagation regimes from the calibration table. In the urban grid, most terrestrial evaluations and approximately 35% of the aerial evaluations are NLoS, for which the calibration prescribes the largest back-off. The highway links are predominantly LoS: the LoS terrestrial links use the RMa K-factor of 7 dB, while the aerial LoS links have a higher median K-factor and consequently receive a smaller calibrated back-off. The mean maximum Doppler frequency of the aerial links is 154 Hz in the urban scenario and 322 Hz on the highway, compared with 141 and 329 Hz for the corresponding terrestrial links. These differences come from the relative speeds and geometries of the two link classes. Their full distributions are given in Section 7.6. These realized operating points are important because $f_{D}$ and *K* are the two channel-state variables that directly determine the proposed correction $\Delta (f_{D},\;K)$. Finally, vehicles changed serving cell approximately 6.2 times per vehicle-minute in the grid and 8.5 times per vehicle-minute on the highway. These rates did not change across feedback settings because the correction does not touch the received-power comparison used for cell selection.

### 7.2. Baseline Results and Interpretation of the Error Metric

Table 9 reports all cells of the factorial design, and Figure 6 summarizes the four network-level KPIs. Two observations provide context for the comparisons that follow. First, with the feedback disabled, the absolute HARQ error rates differ markedly between the scenarios: 18.1 to 18.5% in the urban grid and 2.0 to 2.1% on the highway. The reported metric is the first-transmission error rate of MAC PDUs under Simu5G’s error model. In this model, each resource block contributing to a PDU is decoded independently, and the PDU is lost if any block fails. A PDU spanning *n* blocks with a common per-block BLER *b* is therefore lost with probability $1-{\left(1-b\right)}^{n}$. Supporting diagnostic runs recorded approximately 7 to 10 blocks per PDU in both scenarios, as further discussed in Section 8. The higher urban PDU error rates are therefore consistent with per-block decoding errors adding up across multi-PRB PDUs under mostly NLoS propagation. They should not be read as the per-block BLER target. The absolute rates depend on the configured error model, the propagation conditions, the allocation width and the traffic load. The main comparisons are therefore made within each scheduler, where all of these are held fixed.

Second, the baseline differences among the schedulers are small. In the urban grid, the spread among PF, DRR and MAXCI is below one percentage point for the error rate. The corresponding scheduler effect is F=6.4, compared with F=3019.9 for the feedback factor; the paired differences and complete analysis of variance are reported in Table 10 and Table 11. On the lightly loaded highway, no statistically significant scheduler effect is detected for the error rate (p=0.086). Every vehicle carries one identical CBR flow and there is no downlink traffic, so the schedulers have little traffic diversity to exploit. This helps explain their similar baselines. The paired comparisons between feedback settings within the same scheduler and seed therefore provide the primary evidence for changes associated with the CQI correction.

### 7.3. Effect of the Doppler-Aware CQI Correction

Table 10 reports paired differences between feedback-enabled and feedback-disabled configurations matched by scheduler and seed, while Table 11 summarizes the two-way ANOVA. Figure 7 separates the HARQ first-transmission error rate by serving-cell class and provides the main mechanism-specific evidence. Under A2V-only feedback, the corrected aerial-served class and the uncorrected terrestrial-served class can be compared across the design.

#### 7.3.1. A2V-Only Correction as a Mechanism-Specificity Check

When the correction is enabled only on the aerial-DU links, the first-transmission error rate of the aerial-served vehicles in the urban grid falls from 9.9 to 5.1% (PF), from 10.4 to 5.7% (DRR) and from 10.7 to 4.9% (MAXCI). These changes correspond to relative reductions of approximately 46 to 54%, with the paired comparisons satisfying p≤0.001, while the terrestrial-served rate remains within a fraction of a percentage point of its baseline (Figure 7). This localization is an internal specificity check. The improvement appears in the link class that receives the correction and not in the other class. On the highway, the aerial-served baseline is approximately 0.8% across the three schedulers; A2V-only feedback reduces it to approximately 0.2 to 0.3% (p≤0.002), and it remains within this range when feedback is enabled on all links. The aerial DUs serve 22 to 23% of the urban vehicles, and these vehicles have the lower baseline error rate. The A2V-only setting therefore changes the network-level rate by −1.0 to −1.1 percentage points in the grid (p≤0.035) and by −0.3 to −0.2 percentage points on the highway.

#### 7.3.2. Network-Level Effect of the All-Links Correction

When the correction is enabled on all serving links, the reliability improvement extends to the network. In the urban grid, the network HARQ error rate decreases by 10.6 percentage points under PF (57%), 11.2 under DRR (62%) and 11.2 under MAXCI (60%), all with p<0.001. On the highway, it decreases by 1.1 to 1.2 percentage points (54 to 58%, p≤0.003). The smaller absolute highway improvement is consistent with a floor effect. The baseline error rate is already about 2% at the network level and below 1% for the aerial-served vehicles, so there is less error left to remove.

In the urban grid, most of the network-level improvement comes from the terrestrial links, which are NLoS in approximately 91% of the evaluations and receive the largest calibrated back-off. Depending on the scheduler, their error rate falls from approximately 21% to between 7.5% and 8.7%, corresponding to relative reductions of 59 to 63%. Meanwhile, the aerial-served rate remains close to the corresponding value obtained with A2V-only feedback. On the highway, the terrestrial-served rate similarly falls from approximately 2.8% to between 1.3% and 1.4%.

#### 7.3.3. Comparison with Outer-Loop Link Adaptation

OLLA on all links also lowers the urban network error rate, by 10.3 (PF), 10.2 (DRR) and 10.9 (MAXCI) percentage points (56 to 59%, p<0.001), and it shortens the mean MAC delay by about the same amount as the back-off. The rows labeled “all links vs OLLA” in Table 10 compare the two corrections directly. The back-off gives a lower error rate than OLLA by 0.2 (PF, p=0.305), 1.0 (DRR, p=0.001) and 0.3 (MAXCI, p=0.073) percentage points. The reliability of the two corrections is therefore similar, and the difference is statistically significant only under DRR. The resource cost differs more clearly. OLLA raises PRB utilization by 10.8 to 12.6 percentage points over the baseline, compared with 7.3 to 8.8 points for the back-off. The back-off therefore uses 2.7 to 3.8 percentage points fewer PRBs than OLLA for a similar or lower error rate (p≤0.043 under all three schedulers). No statistically significant throughput difference between the two corrections is detected (p≥0.09). The mean OLLA offset over a run is 4.8 to 5.5 dB on the terrestrial links and 3.0 to 3.2 dB on the aerial links, but its distribution is wide. The terrestrial-link offset has a 25th percentile of −2.5 dB, a median of 7.0 dB and a 90th percentile of 11.2 dB.

On the highway, OLLA does not lower the network error rate. Its paired change relative to feedback off is +0.1 to +0.2 percentage points (p≥0.15), and the mean MAC delay rises by 0.02 to 0.03 ms (p≤0.046). Figure 7 shows why. OLLA moves each UE toward its 10% target from whichever side the UE starts. Most highway UEs start below the target, so their offsets drift to the lower clamp (median −3.0 dB on the aerial links and −2.0 dB on the terrestrial links), and the aerial-served error rate rises from approximately 0.8% to 2.2% under every scheduler. The terrestrial-served rate falls from approximately 2.8% to between 1.9 and 2.2%, which is consistent with OLLA correcting the terrestrial UEs whose error rate lies above the target. Because OLLA behavior depends directly on the selected BLER target, Section 7.7 also evaluates a tighter $t=10^{-2}$ setting to distinguish target-selection effects from differences between the two correction mechanisms. The proposed back-off does not adapt toward a fixed ACK/NACK-based BLER target; instead, it applies the channel-state-dependent correction calibrated from $f_{D}$ and *K*, and in these highway runs it lowers the error rate of both serving classes. Relative to OLLA it reduces the network rate by 1.2 to 1.3 percentage points (56 to 62%, p<0.001), with no statistically significant PRB or throughput difference.

The feedback main effect has the largest ANOVA *F* statistic for the error rate in both scenarios (F=3019.9 urban and F=98.8 highway; p<0.001 in both cases). The scheduler main effect is much smaller: it is statistically significant in the urban grid (F=6.4, p=0.003) but not on the highway (F=2.6, p=0.086). No statistically significant scheduler-by-feedback interaction is detected in either scenario (p=0.156 urban and p=0.938 highway), providing no evidence in these runs that the effect of the correction depends on the evaluated scheduler.

### 7.4. Reliability–Resource Trade-Off

In the urban grid, the mean MAC delay decreases by 0.35 to 0.38 ms across the three schedulers, with p<0.001 in all cases. The share of PDUs delivered on the first transmission rises from 78.2 to 89.2% under PF, from 79.2 to 90.6% under DRR and from 77.9 to 89.4% under MAXCI. These reliability gains use more resources. They should therefore be read as a trade-off between reliability and resource efficiency, not as a cost-free improvement. The more conservative MCS selections increase PRB utilization by 7.3 to 8.8 percentage points, with p<0.01 for all schedulers. By contrast, the signed changes in per-UE throughput range from −2.2 to −1.2 kb/s and are not statistically significant under any scheduler (p≥0.09).

On the lightly loaded highway, where baseline PRB utilization is approximately 4.1% and 97.7% of the PDUs already succeed on the first attempt, the mean delay decreases by 0.02 to 0.03 ms and the first-transmission share rises by approximately 0.7 to 1.0 percentage points. These changes are statistically significant only under MAXCI, for which throughput also increases slightly; PRB utilization does not change significantly under any scheduler. The wide highway throughput intervals in Figure 6 follow from the KPI definition in Appendix A. Only about 25 vehicles are present at a time and they enter at different times, so the equal-weight per-UE average varies substantially between seeds. Throughput is therefore interpreted as a supporting KPI rather than as the primary endpoint. Overall, the results are consistent with the reliability-oriented back-off of Section 4.5, which trades wider initial allocations for fewer retransmissions. Section 7.7 examines how this balance changes with the scale α. OLLA on all links reaches the same delay and first-transmission share as the back-off in the urban grid (Table 9) but at 2.7 to 3.8 percentage points higher PRB utilization (Section 7.3).

### 7.5. Delay Distributions

Figure 8 shows the empirical cumulative distribution function (CDF) of the uplink MAC delay per PDU, reconstructed from the fixed-bin histograms recorded by the RIC using 0.1 ms bins. Each panel overlays, for the PF scheduler, the four feedback settings and, for the feedback-off case, the aerial-served and terrestrial-served subsets, so that the reader can see where the delay mass sits and which subset forms the tail. Because Simu5G schedules uplink transmissions on a 1 ms TTI grid and retransmits after a fixed HARQ round trip, the delays are concentrated at a few discrete values: approximately 2 ms for a PDU delivered on the first transmission and an additional 3 ms for each retransmission. The 0.1 ms histogram binning places the reported upper edges of these delay steps at 2.1, 5.1 and 8.1 ms.

The resulting distributions are therefore staircases, and a percentile remains on the same step across many configurations. The 95th percentile is 5.1 ms in every urban configuration and 2.1 ms in every highway configuration. The 99th percentile is 8.1 ms in the urban scenario and 5.1 ms on the highway in all but two configurations, for which some seeds place it one step lower. For comparisons among feedback settings, the height of the first step is more informative than the position of these upper percentiles because it represents the first-transmission share reported in Table 9. A percentile changes only when the probability mass crosses a step boundary, whereas the mean delay and first-transmission share capture smaller shifts in the mass assigned to each step. They therefore respond more finely to the feedback effect and are used as the supporting indicators in Section 7.4. The figure shows a shift of probability mass from the second step (one retransmission) to the first step (no retransmission) when a correction is enabled, while the positions of the steps do not change. In the urban grid the OLLA curve lies on top of the all-links curve. On the highway its first step is lower than that of the feedback-off curve (97.1% against 97.7% of PDUs delivered on the first transmission), which is the delay-side view of the error-rate increase reported in Section 7.3. What follows from this is that the corrections reduce the number of retransmissions rather than the duration of each one. This is the behavior expected from a correction that acts on the choice of modulation and coding scheme.

The class-specific curves show that the urban delay tail is associated primarily with the terrestrial-served PDUs. With feedback disabled under PF, 84.3% of aerial-served PDUs and 76.5% of terrestrial-served PDUs are delivered on the first transmission. The A2V-only setting increases the aerial-served share to 88.8%, while the terrestrial-served share remains at 76.3%. Enabling feedback on all links increases the corresponding shares to 88.0% and 89.5%, respectively. On the highway, 99.2% of aerial-served PDUs and 96.5% of terrestrial-served PDUs succeed on the first transmission without the correction; all-links feedback increases the terrestrial-served share to 97.3%.

### 7.6. Recorded A2V Observables

Figure 9 shows the pooled distributions of the six quantities recorded by the A2V module at each channel evaluation of a served aerial link across all factorial runs. These distributions describe the operating conditions seen by the correction, not its performance. Table 12 summarizes them numerically so that the two scenarios can be compared directly. The same trajectories are pooled across treatments, so the distributions are read descriptively and are not used as independent samples for statistical tests.

The maximum Doppler frequency spans approximately 63.0 to 243.0 Hz from the 10th to the 90th percentile in the urban grid and 148.3 to 524.6 Hz on the highway. In the highway scenario, a UAV flying along the road encounters vehicles traveling in the same direction at relatively low differential speeds and vehicles traveling in the opposite direction at relative speeds approaching 44.9 m/s at the 90th percentile. The bimodal highway distribution is consistent with these same-direction and oncoming populations, although the recorded samples are not classified by travel direction. The LoS Doppler shift is nearly symmetric around zero in the urban grid, where vehicles approach and recede along different directions, but is negatively skewed on the highway, with a median of −120.0 Hz. Because travel direction is not recorded with each sample, the source of this asymmetry cannot be established from these observations alone.

The median elevation angles are $38.1^{\circ }$ in the urban grid and $52.0^{\circ }$ on the highway. For LoS links, the Rician *K*-factor is calculated from the elevation angle using Equation (6); its recorded 10th- to 90th-percentile range is 8.0 to 18.0 dB in the grid and 8.9 to 20.4 dB on the highway. The computed back-off is bimodal in the urban grid. It is concentrated around 7.5 to 7.9 dB for the NLoS third of the aerial links, corresponding to the Rayleigh row of Table 3, and is mostly below 3 dB for the LoS links, with an overall median of 1.3 dB. On the highway, it is generally smaller, with a median of 1.0 dB and a 90th percentile of 3.3 dB. The candidate back-off is computed and recorded under every feedback setting. The switch only decides whether it is subtracted before CQI quantization. The relative-speed distributions span 5.5 to 20.7 m/s in the urban grid and 12.4 to 44.9 m/s on the highway.

The recorded ICI penalty, which is not plotted in Figure 9, provides an additional operating-regime indicator. Its median is 0.2 dB in the urban grid and 1.7 dB on the highway, with 90th percentiles of 2.9 and 6.7 dB, respectively. These values arise when LoS Doppler shifts of several hundred hertz coincide with SINRs above 30 dB on the short aerial links. The penalty limits the SINR of the strongest links to approximately 30 dB and is included identically in the feedback and data paths. It therefore does not differ between the feedback settings. The reported *K*-factor and Doppler distributions therefore represent the realized operating points at which the channel-state-dependent correction was evaluated, rather than nominal scenario parameters.

### 7.7. Sensitivity Analysis

Figure 10 reports the sensitivity analysis for the highway scenario under PF scheduling. Each parameter level uses three seeds. This analysis therefore gives secondary evidence about the behavior of the correction, not the confirmatory evidence of the five-seed factorial experiment. Moreover, most highway links operate in the LoS, low-back-off regime and have low baseline error rates. The results should therefore be interpreted as trends over the tested parameter ranges rather than precise estimates of the corresponding effects.

Back-off scale α. The network HARQ error rate decreases monotonically as the back-off is scaled from α=0 (1.8%) through 0.5 (1.2%), 1 (0.9%) and 1.5 (0.6%) to 2 (0.6%), with the final two levels forming an apparent plateau. Over the same range, the terrestrial-served error rate decreases from 2.5% to 1.0%, and the aerial-served rate from 0.7% to 0.1%. Per-UE throughput changes from 40.7 to 39.6 kb/s and mean delay from 2.06 to 2.05 ms, without a systematic change beyond the seed-to-seed spread. In this scenario, the calibrated value α=1 appears as an intermediate point on a smooth reliability curve rather than as a distinct knee. The urban PRB results suggest that increasing α under heavier load would incur a greater resource cost.

Rician *K*-factor. Raising $K_{1}$ from 1 to 4 increases the Rician *K*-factor at the representative highway elevation angle of $52^{\circ }$ from 11 to 17 dB. The mean computed back-off for the aerial links consequently decreases from 2.6 to 0.9 dB, while the aerial-served error rate with feedback disabled decreases from 2.3% to 0.3%. With feedback enabled, the corresponding rates are 0.9%, 0.3% and 0.1% at $K_{1}=1$, 2 and 4, respectively. Both the loss associated with CSI aging and the improvement produced by the correction therefore decrease as *K* increases, consistent with the calibration in Table 3. The terrestrial links remain unaffected because $K_{1}$ is used only by the A2V channel model.

CQI reporting period. Changing the CQI reporting period from 2 TTIs (report age of 1 to 3 ms) to 6 TTIs (1 to 7 ms) and 10 TTIs (1 to 11 ms) produces no monotonic change in the baseline error rate. The respective rates are 2.2%, 1.8% and 2.0% with feedback disabled, compared with 1.0%, 0.9% and 0.9% with feedback enabled. At the observed highway Doppler frequencies, including a median aerial-link maximum Doppler of 232.8 Hz, the diffuse channel component decorrelates within a few milliseconds. This rapid decorrelation is consistent with the weak sensitivity observed across the three report-age ranges.

Vehicle-speed scale. Scaling the vehicle speeds to one half and three quarters of their nominal values reduces the median aerial-link maximum Doppler to approximately 130 and 226 Hz, respectively. Nevertheless, the baseline error rates show no monotonic dependence on speed: they are 2.1%, 2.3% and 1.8% at half, three-quarter and full speed. With feedback enabled, the corresponding rates are 1.1%, 1.1% and 0.9%, representing an absolute reduction of approximately one percentage point at each level. The weak dependence over this range is consistent with operation in the saturation region of the calibrated back-off, above approximately 100 Hz of maximum Doppler. These results support robustness over the tested speed range but should not be interpreted as evidence of general speed independence.

OLLA target. The OLLA arm of the factorial experiment uses the conventional 10% target. To see how much the choice of target matters, the 30 runs of that arm were repeated with the tight target $t=10^{-2}$ (five seeds, both scenarios, all three schedulers). In the urban grid the offset then drifts toward its upper clamp (mean 9.6 to 9.8 dB on the terrestrial links), and the network error rate falls to 4.7 to 5.4%, below the back-off by 2.3 to 2.6 percentage points. PRB utilization, however, rises to 51.5 to 54.7%, that is 6.4 to 9.3 points more than with the back-off (all p≤0.002), with no statistically significant throughput difference. On the highway the tight target gives a network error rate of 0.9 to 1.0%, not significantly different from the back-off (p≥0.5). The target therefore sets where OLLA lands on the reliability–resource curve. The proposed back-off therefore occupies an intermediate point on the tested reliability–resource trade-off without requiring online ACK/NACK-based target tracking or error-history convergence.

## 8. Discussion

The experimental results validate that the proposed Doppler-aware CQI feedback correction consistently reduces the Hybrid Automatic Repeat Request (HARQ) first transmission PDU error rate across all three MAC scheduling algorithms and both evaluation topologies. The A2V-only configuration confirms that performance gains remain confined to the specific link category receiving the correction. Paired statistical comparisons further isolate these gains from random seed-dependent traffic and small-scale channel variations. Overall gain magnitude is larger under lower Line-of-Sight (LoS) availability and lower Rician *K*-factor, whereas the associated physical resource block (PRB) overhead is governed by carrier load. When evaluated head-to-head against outer-loop link adaptation (OLLA) using the main target of t=0.1, the proposed scheme achieves comparable or lower first-transmission PDU error rates while using fewer radio resources in urban settings and achieves lower first-transmission PDU error rates under highway conditions.

### 8.1. Residual PDU Errors

While the Doppler-aware correction substantially lowers first transmission PDU errors, it does not eliminate them entirely. With feedback enabled across all links, the residual urban first-transmission PDU error rate stabilizes between 5.0% and 5.5% for aerial-served UEs and between 7.5% and 8.7% for terrestrial-served UEs; under highway conditions, the overall network error rate settles at approximately 1.0%. This behavior aligns with theoretical expectations: the SINR back-off is calibrated to restore baseline per-block BLER dynamics under CSI aging, rather than aggressively forcing the multi-block PDU error rate down to a strict $10^{-2}$ target. Integer-decibel CQI quantization leaves a residual per-block BLER between 1.5% and 3.5%, which compounds exponentially over typical 7 to 10 PRB allocations. Additionally, newly handed-over vehicles may be scheduled before their target cell receives a refreshed, valid CQI report. Supporting single-seed diagnostics on urban terrestrial links confirm that the per-block BLER decreases from 6% to 2% while the average allocation widens from 7 to 8 PRBs, consistent with the PDU-level error reduction from 21% to 9%.

### 8.2. Propagation Regime Dependence

The system-level evaluations mirror the link-level calibration trends. In the urban grid topology, about 91% of terrestrial link instances and 35% of aerial link instances operate under NLoS conditions and therefore receive the maximum calibrated SINR back-off. As a result, the correction yields substantial reliability enhancements across both serving tiers. Conversely, highway propagation is predominantly LoS, with Rician *K*-factors spanning 7 to 20 dB, resulting in milder SINR adjustments. Although absolute percentage-point reductions on the highway appear smaller due to a low baseline error rate (∼2%), the relative error reduction remains comparable to that of the urban grid. Thus, the two scenarios provide complementary evaluations: the urban grid evaluates performance in NLoS-heavy fading environments, while the highway scenario tests system resilience under high mobility and strong LoS propagation.

### 8.3. Reliability vs. Resource Overhead

The pre-quantization back-off prioritizes first-transmission reliability over absolute throughput maximization. In urban grid deployments, suppressing HARQ retransmissions decreases mean MAC layer latency by 14 to 15% at the cost of increasing PRB utilization by 7.3 to 8.8 percentage points. Per-UE throughput shows no statistically significant change, demonstrating that the correction trades wider initial bandwidth allocations for fewer retransmission cycles. On the lightly loaded highway topology where baseline PRB utilization is only 4.1% and 97.7% of PDUs succeed on the first attempt, latency, throughput, and resource variations remain minimal. Consequently, the scaling parameter α serves as an explicit tuning knob to balance reliability against spectral efficiency.

### 8.4. Benchmark Comparison with OLLA

Head-to-head performance comparisons reveal two critical architectural properties of the proposed scheme relative to conventional OLLA:

Target-Free Operation: OLLA operates as a closed-loop set-point controller, driving the error rate toward a target threshold regardless of initial link conditions. In urban scenarios where baseline error rates exceed the 10% target, OLLA and the proposed SINR back-off achieve comparable error rates and latencies; however, OLLA consumes 2.7 to 3.8 percentage points higher PRB utilization. On the highway where average error rates start below target, OLLA inadvertently degrades aerial-served link reliability and fails to improve network-wide error rates. In contrast, the Doppler-aware back-off applies an explicit SINR margin derived from CSI aging dynamics, applying strong corrections under rapid fading (NLoS, high Doppler) and minimal adjustments under stable propagation (LoS, high *K*-factor).

Convergence & Bimodal Distribution: Recorded OLLA offsets exhibit high variance and bimodal distributions. In urban terrestrial links, the median OLLA offset is 7.0 dB, but spans a 25th percentile of −2.5 dB to a 90th percentile of 11.2 dB. Aerial-link OLLA offsets frequently saturate near upper or lower bounds (median −2.4 dB, 75th percentile 11.2 dB). UEs clamped near the upper offset limit are assigned conservative MCS levels, driving excessive PRB utilization. Furthermore, OLLA depends on historical ACK/NACK feedback which is unavailable immediately following a handover and adapts incrementally per frame. The proposed back-off tracks real-time Doppler frequency and Rician *K*-factor within a single reporting interval.

### 8.5. Aerial-Tier Association Dynamics

Under the configured RSSI-based cell selection with hysteresis, aerial distributed units (DUs) serve 22 to 23% of active vehicles in the urban grid and approximately 45% in the highway scenario, with handover rates of 6.2 and 8.5 handovers per vehicle-minute, respectively. Holding these association parameters constant isolates the explicit performance impact of the CQI correction. Advanced mobility management features such as time-to-trigger (TTT) windows [29] or aerial-tier cell-selection offsets [30] can be layered onto this framework without altering the underlying feedback dynamics.

### 8.6. Limitations and Future Work

This study evaluates the RAN Intelligent Controller (RIC) as an integrated measurement plane while isolating uplink CSI aging under controlled conditions, operating xApps in shadow mode. Inter-cell interference and downlink data traffic are omitted, while aerial backhaul is idealized, in order to isolate the core feedback mechanism. Future extensions will incorporate closed-loop near-RT RIC xApp control, multi-cell interference mitigation, and realistic aerial backhaul constraints. Additionally, over-the-air field measurements will validate channel parameterization under real-world hardware and feedback-channel impairments.

## 9. Conclusions

This paper presented a Doppler-aware CQI feedback correction mechanism designed for 3D dual-mobility Air-to-Vehicle (A2V) links within an Open RAN framework implemented in Simu5G. By modeling joint 3D mobility between UAV aerial small cells and mobile ground vehicles, the framework exposes real-time channel state parameters and performance metrics over an O-RAN-based measurement plane. The proposed scheme mitigates mobility-induced CSI aging by subtracting a calibrated SINR offset parameterized by maximum Doppler frequency and channel Rician *K*-factor prior to CQI quantization.

Multi-factorial experimental evaluations across three MAC schedulers, four feedback configurations, two propagation topologies, and five paired random seeds demonstrate the following key findings:

- Targeted A2V Improvement: Restricting the correction to aerial-DU links reduces the first-transmission HARQ error rate for aerial-served UEs by 46 to 54% in urban grids and from approximately 0.8% down to 0.2–0.3% on highways, without affecting terrestrial link performance.
- Network-Wide Impact: Applying the correction network-wide reduces overall first-transmission PDU error rates by 57 to 62% in urban grids and 54 to 58% on highways across all tested schedulers, with no statistically significant interaction between the scheduler choice and feedback correction performance.
- Latency and Overhead: In urban scenarios, the scheme improves first-transmission success rates by 11.0 to 11.5 percentage points and cuts mean MAC delay by 0.35 to 0.38 ms, requiring an additional 7.3 to 8.8 percentage points of PRB utilization with no statistically significant change in per-UE throughput.
- Comparison with OLLA: Compared with the benchmark OLLA configuration using t=0.1, the proposed scheme achieves equal or lower error rates in urban grids using 2.7 to 3.8 percentage points less PRB overhead, while reducing highway network error rates by an additional 1.2 to 1.3 percentage points.

The simulation codebase, configuration files, calibration algorithms, and execution logs will be open-sourced to support reproducibility and future research. Overall, the Doppler-aware CQI correction provides a history-independent, spectrally efficient solution for link adaptation in high-mobility 3D aerial–terrestrial networks.

## Figures and Tables

**Figure 1:**
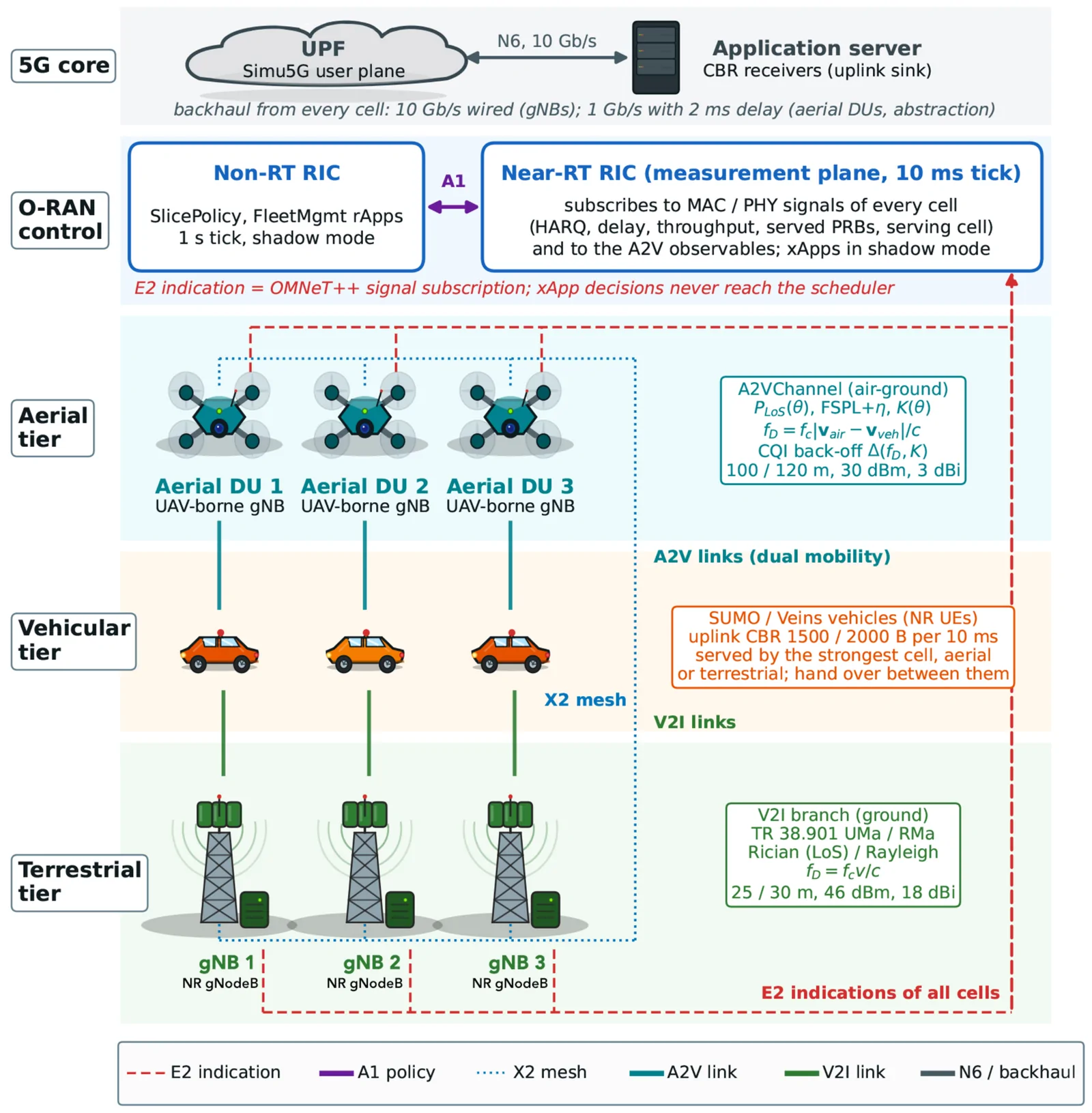
The implemented three-tier air–ground framework; the vehicular tier is drawn between the two cell tiers it hands over between. Vehicles (Simulation of Urban MObility (SUMO)/Veins) are NR user equipment served either by a UAV-borne aerial distributed unit (A2V link, dual mobility) or by a terrestrial gNB (V2I link). All cells share an X2 mesh and a backhaul to the user plane function (UPF) and the application server. The Near-RT RIC is a measurement plane: it subscribes to the MAC/PHY signals of every cell and to the A2V channel observables (E2 indication emulated by an OMNeT++ signal subscription); the xApps and the Non-RT RIC rApps run in shadow mode and do not alter the scheduler.

**Figure 2:**
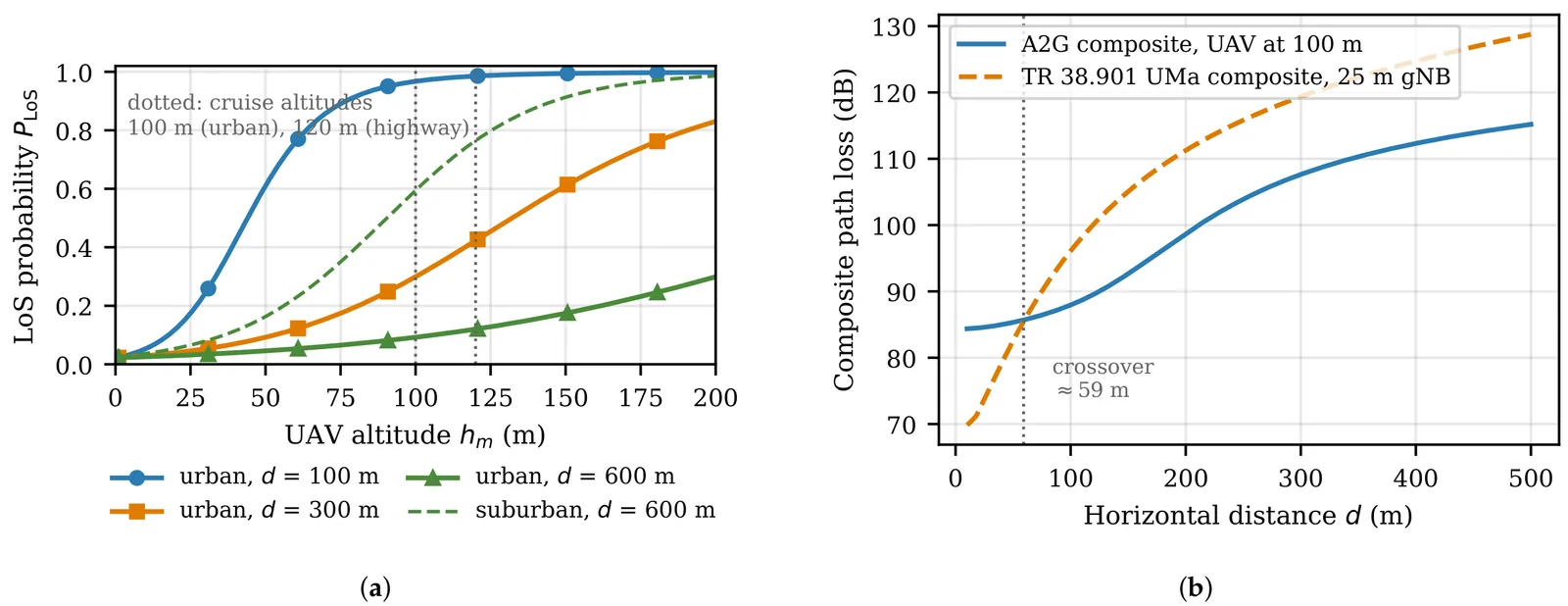
Large-scale A2G geometry, evaluated from the model equations (not simulation output). (**a**) $P_{\mathrm{LoS}}$ of Equation (3) versus altitude for horizontal distances of 100, 300 and 600 m (urban parameters). (**b**) Composite A2G loss of Equation (5) at 100 m altitude against the TR 38.901 UMa composite for a 25 m gNB.

**Figure 3:**
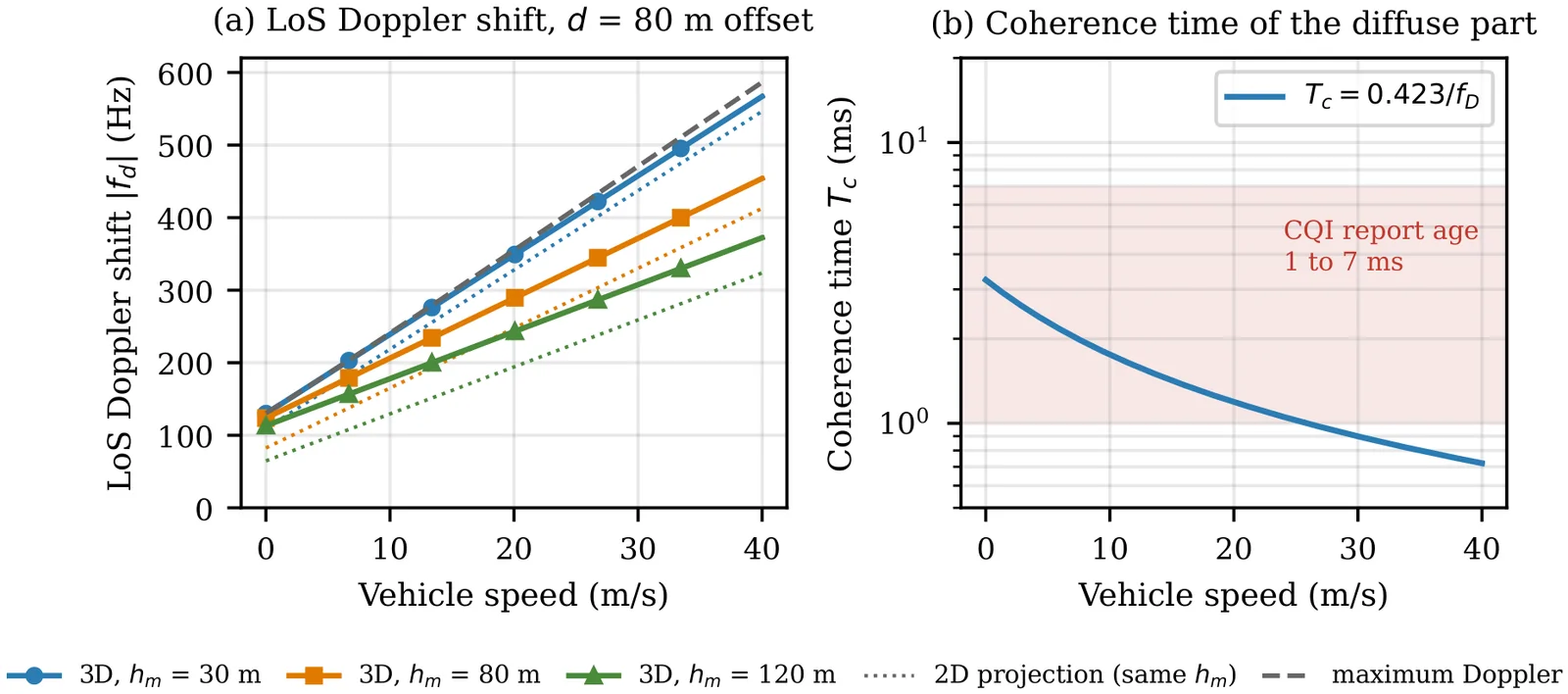
(**a**) Doppler shift of the LoS component, Equation (2), against the vehicle speed at 3.5 GHz for a UAV that flies at 10 m/s against the vehicle while descending at 5 m/s, at a horizontal offset of 80 m and three altitudes; dotted lines drop the vertical terms (2D projection), the dashed line is the maximum Doppler $f_{D}$ of Equation (1). (**b**) Coherence time $0.423/f_{D}$ of the diffuse component; the shaded band is the age of a CQI report when it is used. Both panels are evaluated from the equations.

**Figure 4:**
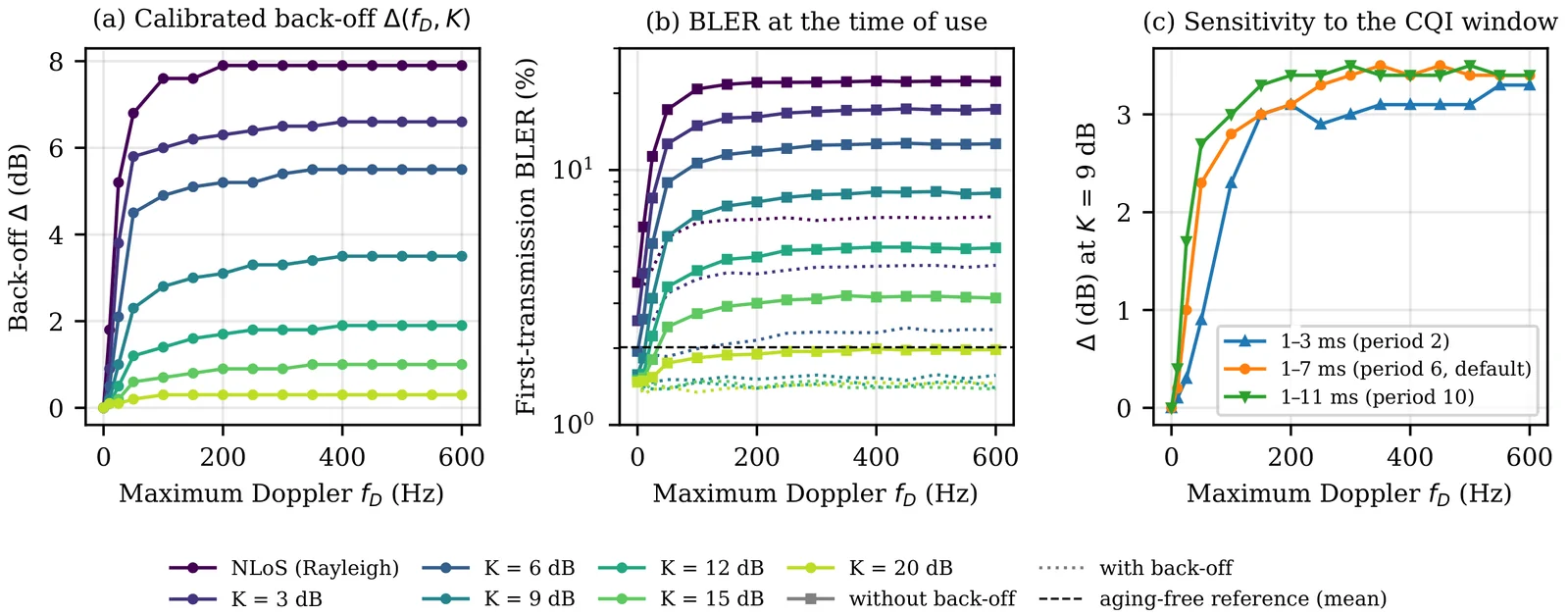
Link-level calibration of the back-off. (**a**) $\Delta (f_{D},K)$ from Table 3. (**b**) Realized first-transmission BLER at the time of use without the back-off (solid) and with it (dotted); the dashed line marks the mean aging-free reference. (**c**) Sensitivity of Δ to the CQI window for K=9 dB (report ages of 1 to 3, 1 to 7 and 1 to 11 ms).

**Figure 5:**
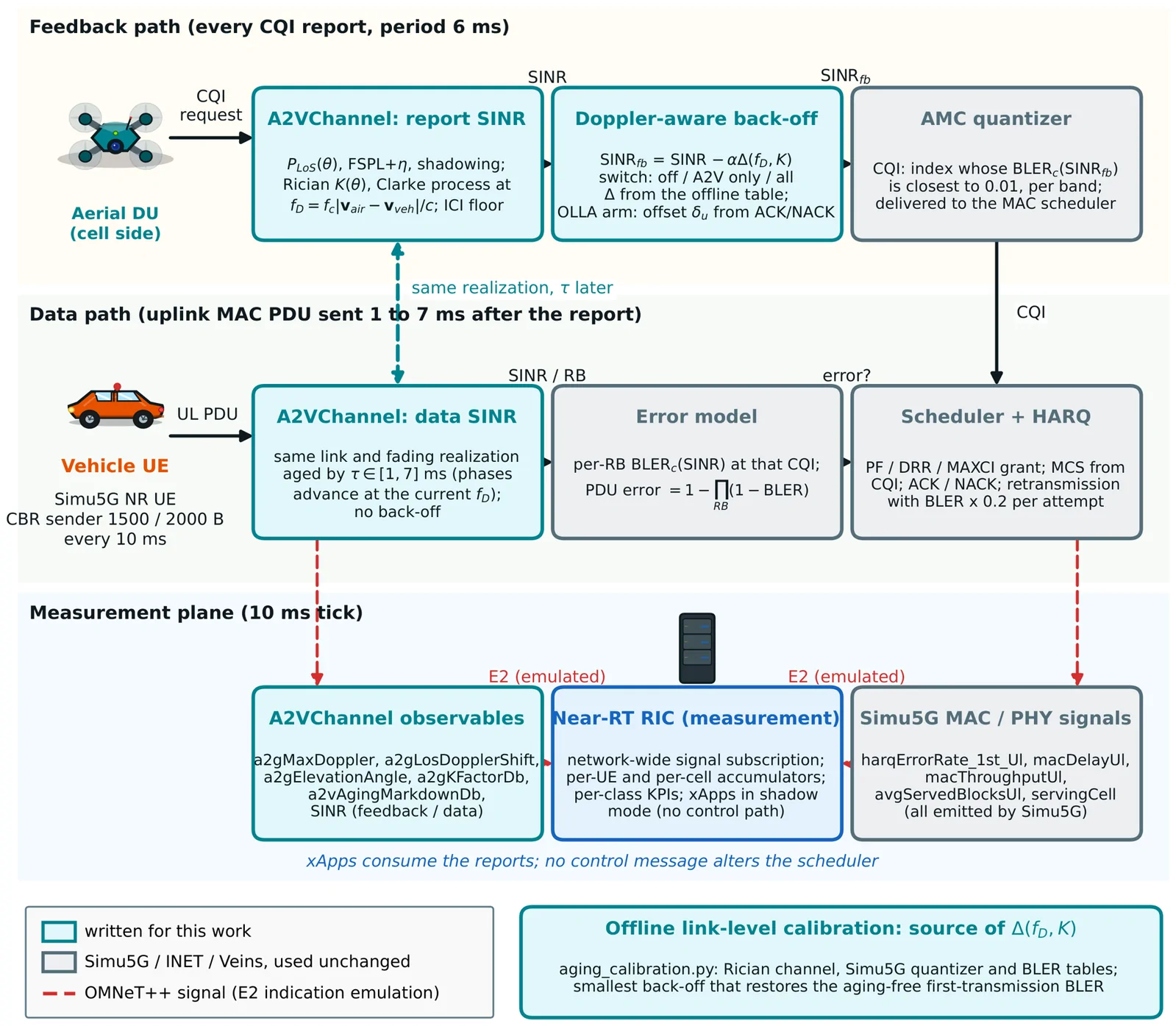
Processing path used to evaluate the proposed CQI correction and the OLLA comparison arm. Teal boxes denote components implemented for this work, whereas gray boxes denote Simu5G modules used unchanged. Signal names correspond to the identifiers used in the implementation and recorded result files; a2vAgingMarkdownDb is the internal name of the applied CSI-aging back-off Δ defined in Equation (8). The effective CQI is formed in the feedback path of the aerial DU: the A2V channel module provides the measured SINR, after which either the proposed back-off $\Delta (f_{D},K)$ or, in the comparison arm, the OLLA offset $\delta_{u}$ is applied at the same pre-quantization point. Simu5G’s unchanged quantizer then maps the resulting SINR to a CQI. The data path uses the same fading realization, aged by the CQI report delay, without applying either correction to the data-path SINR.

**Figure 6:**
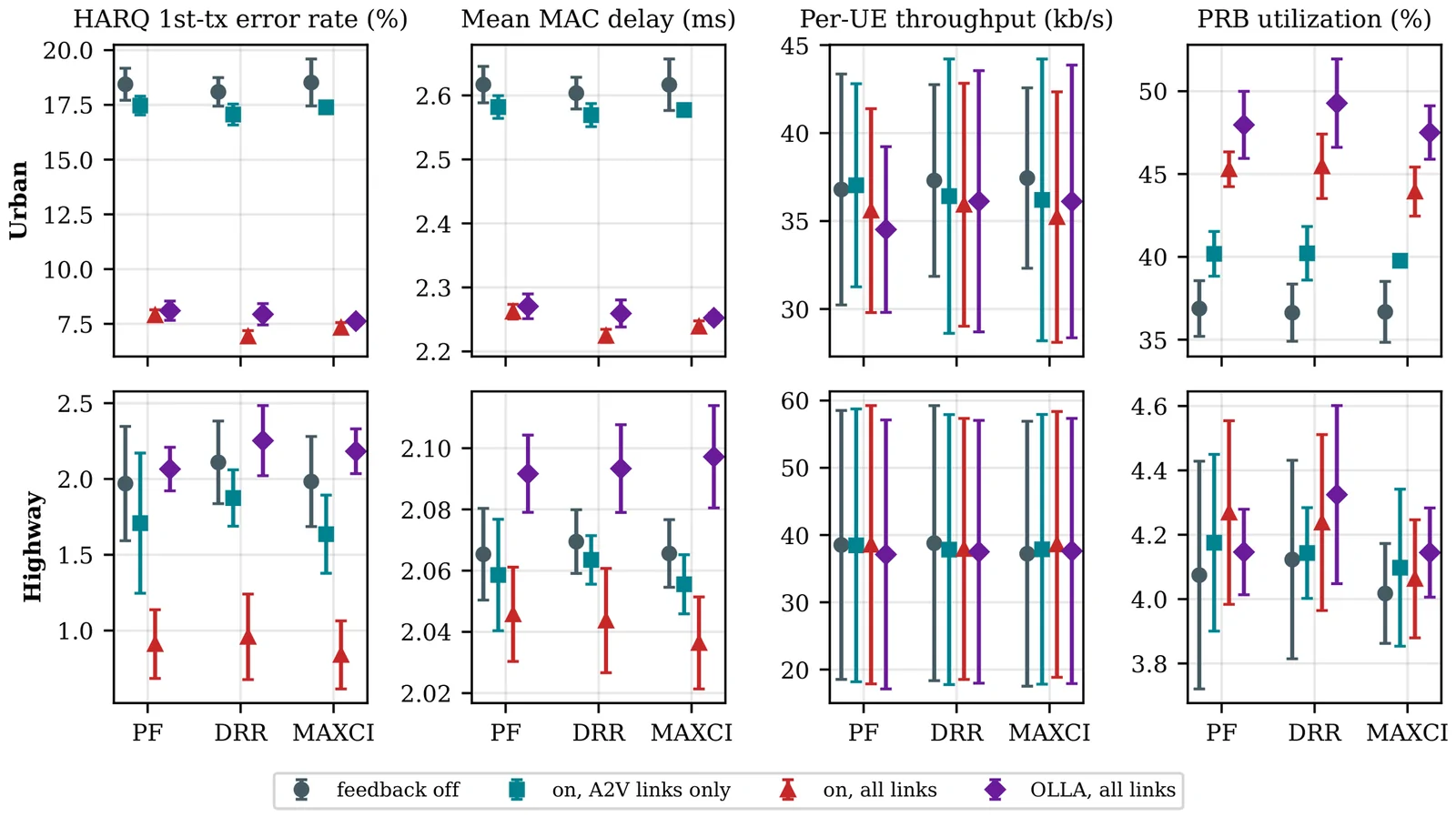
Network-level KPIs for the factorial design across the three schedulers and four feedback settings (off, back-off on A2V links only, back-off on all links, and the OLLA comparison arm). The points show the means over five seeds, and the error bars show the 95% confidence intervals; the top row presents the urban scenario and the bottom row the highway scenario.

**Figure 7:**
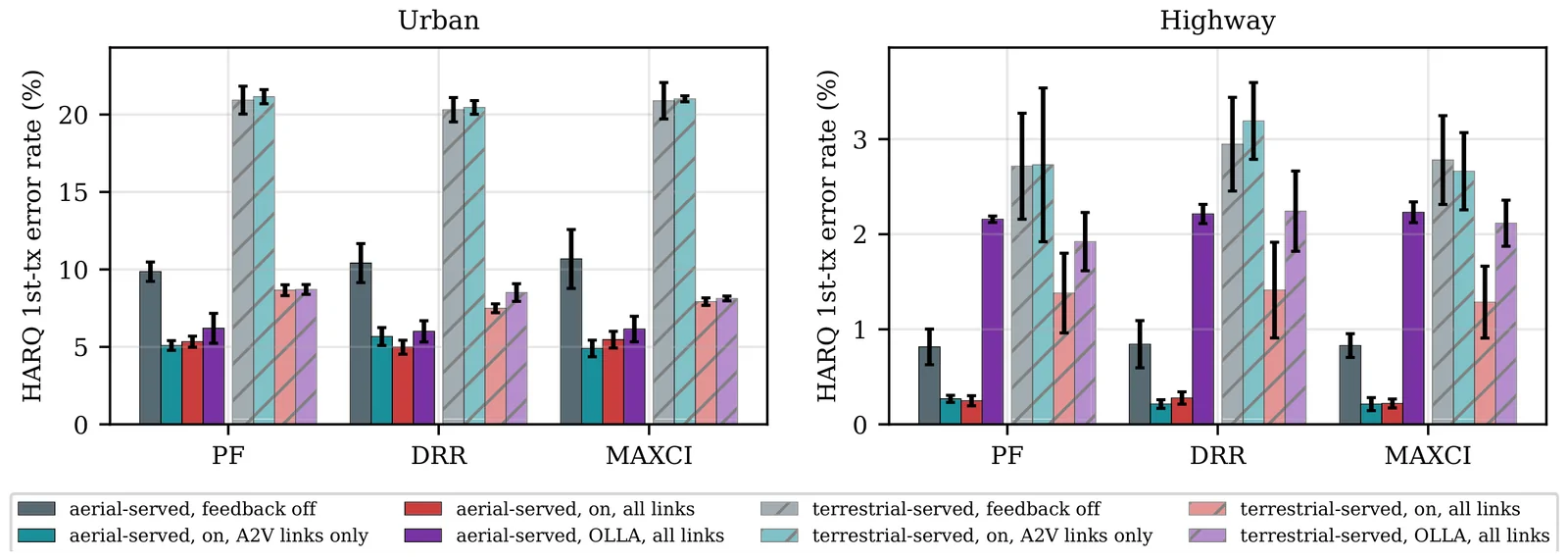
HARQ first-transmission error rate of the aerial-served (solid) and terrestrial-served (hatched) vehicles for every cell of the design (mean and 95% CI over five seeds; note the different ordinate scales). The A2V-only setting reduces the aerial-served rate while the terrestrial-served rate remains near its baseline; the all-links setting and the OLLA arm act on both classes.

**Figure 8:**
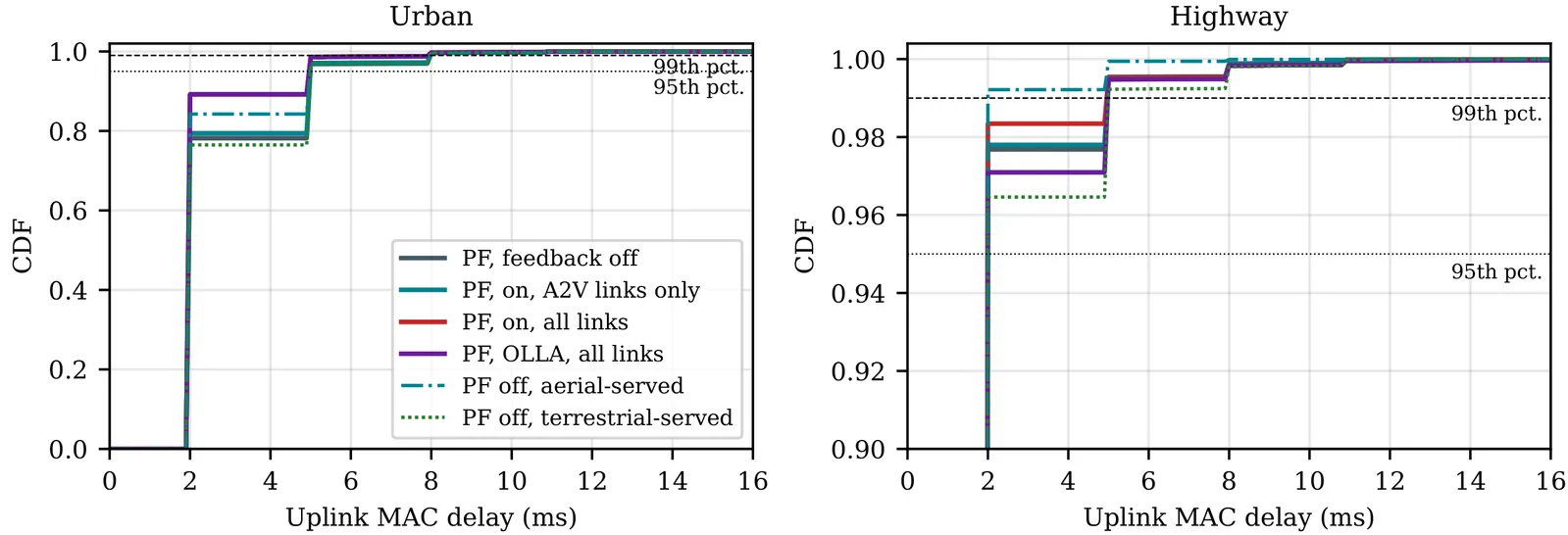
Empirical CDFs of the uplink MAC delay per PDU, reconstructed from the recorded 0.1 ms histograms under the PF scheduler. The curves show the four network-level feedback settings and the aerial-served and terrestrial-served subsets with feedback disabled; left, urban scenario; right, highway scenario, with the ordinate restricted to 0.9–1. The dotted and dashed horizontal lines mark the 95th and 99th percentiles.

**Figure 9:**
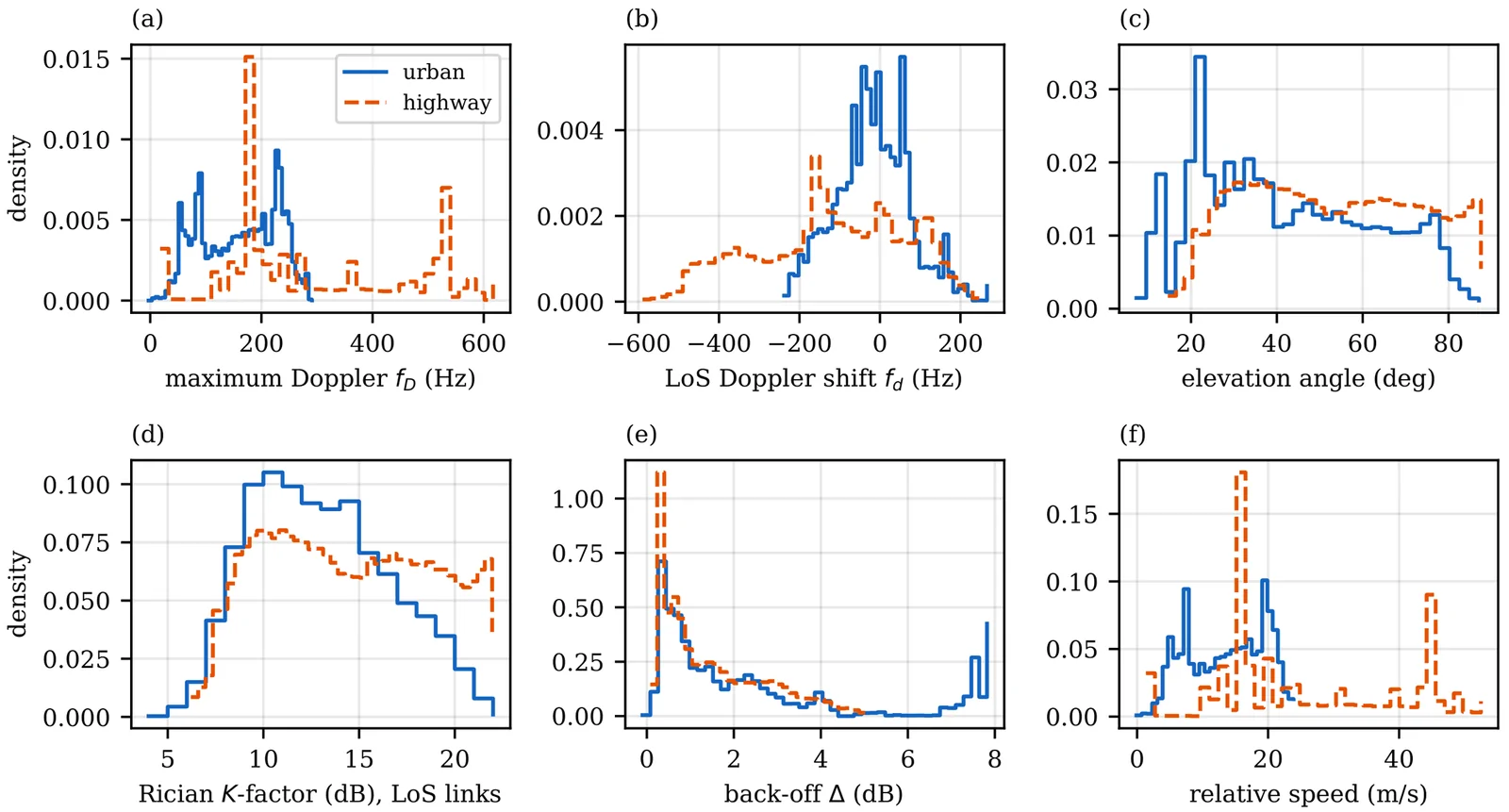
Recorded distributions of the A2V observables for the aerial DUs, pooled across all factorial runs: (**a**) maximum Doppler frequency $f_{D}$; (**b**) LoS Doppler shift $f_{d}$; (**c**) elevation angle; (**d**) Rician *K*-factor of the LoS links; (**e**) computed back-off Δ; and (**f**) relative speed. The vertical axes show density per unit of the corresponding horizontal-axis variable.

**Figure 10:**
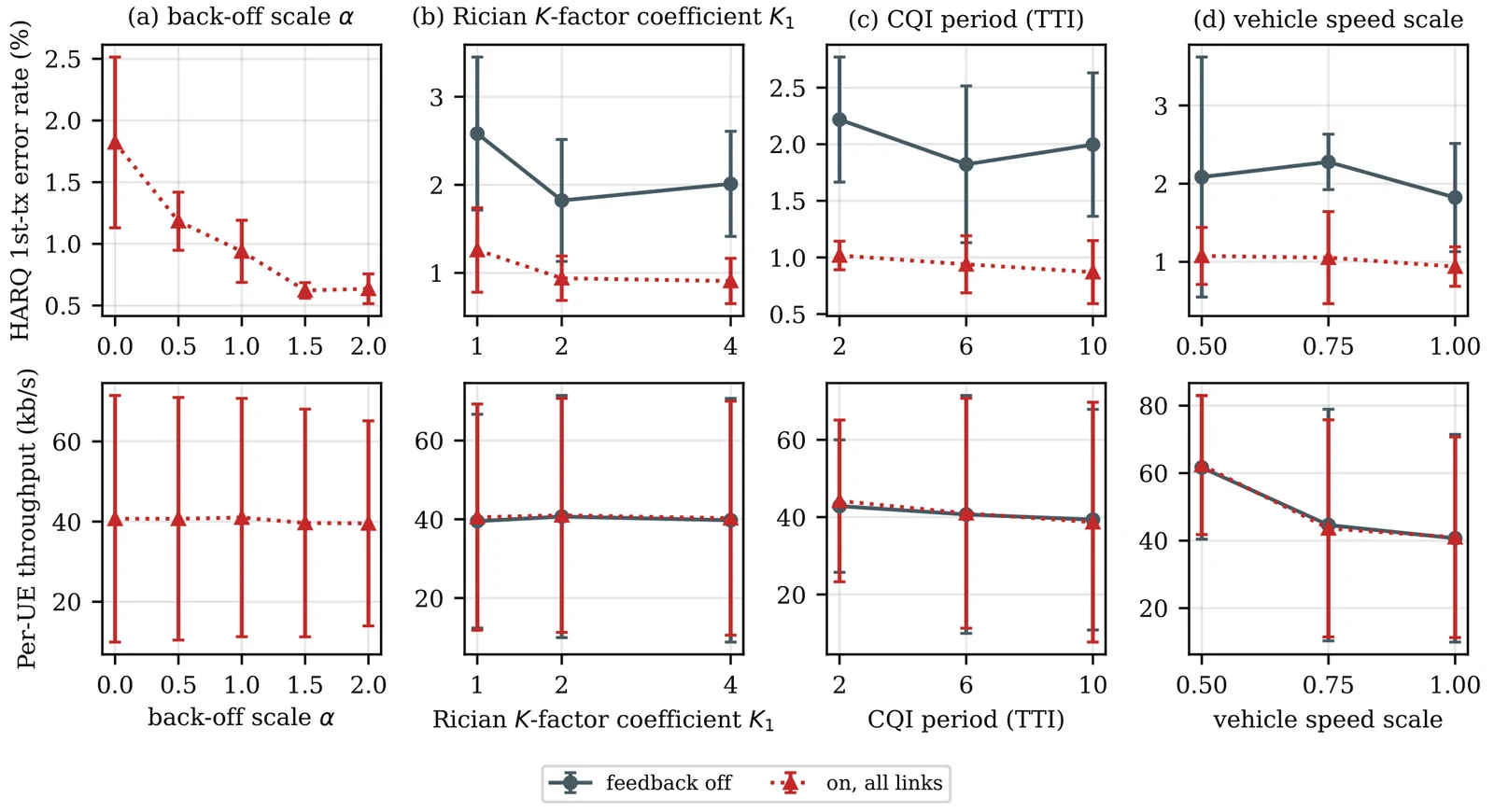
Sensitivity analysis for the highway scenario under PF scheduling. Points show the means over three seeds per level, and error bars show the 95% confidence intervals. The top row presents the network HARQ first-transmission error rate and the bottom row the per-UE throughput as functions of (**a**) the back-off scale α, (**b**) the Rician *K*-factor coefficient $K_{1}$, (**c**) the CQI reporting period and (**d**) the vehicle-speed scale. Feedback is enabled on all links in panel (**a**), while panels (**b**–**d**) compare feedback disabled with feedback enabled on all links.

**Table 1:** Positioning of the closest prior work. ✔ = addressed; × = not addressed. UAV mob. = 3D UAV mobility; Veh. = vehicular ground nodes; Dual mob. = both endpoints mobile; Simu5G = Simu5G integration; Fb. eval. = controlled evaluation of a channel-aware feedback correction; vs. OLLA = direct comparison with outer-loop link adaptation.

Reference	UAV Mob.	Veh.	Dual Mob.	Simu5G	Fb. Eval.	vs. OLLA
Al-Hourani et al. [10]	×	×	×	×	×	×
Matolak and Sun [12]	×	×	×	×	×	×
Bian et al. [14]	✔	×	×	×	×	×
Bai et al. [15]	✔	×	×	×	×	×
Kavas-Torris et al. [16]	×	✔	✔	×	×	×
Moro et al. [22]	×	✔	×	×	×	×
Sroka and Kliks [23]	×	✔	×	×	×	×
This work	✔	✔	✔	✔	✔	✔

**Table 2:** Main symbols. Units in parentheses; dB quantities are power ratios.

Symbol	Meaning
q, p; $v_{a},\;v_{g}$	position and velocity of the aerial and the ground node (m; m/s)
$d_{3D}$, $d_{2D}$, θ	3D distance, horizontal distance (m) and elevation angle (degrees) of the link
$v_{\mathrm{rel}}$, $\hat{d}$	relative velocity $v_{a}-v_{g}$ and unit vector from aerial to ground node
$f_{c}$, *c*, Δf	carrier frequency (3.5 GHz), speed of light, subcarrier spacing (30 kHz)
$f_{D}$, $f_{d}^{\mathrm{LoS}}$	maximum Doppler frequency and LoS Doppler shift of the link (Hz), Equations (1) and (2)
$P_{\mathrm{LoS}}\left(\theta \right)$; a, b	LoS probability, Equation (3), and its environment parameters
PL, $\bar{\mathrm{PL}}$	path loss of one LoS state and its LoS-averaged composite (dB), Equations (4) and (5)
$\eta_{\mathrm{LoS}},\;\eta_{\mathrm{NLoS}}$	mean excess loss over free space in the LoS and NLoS state (dB)
$\sigma_{\mathrm{LoS}},\;\sigma_{\mathrm{NLoS}}$	standard deviation of the log-normal shadowing in the LoS and NLoS state (dB)
*K*; $K_{1},\;K_{2}$	Rician K-factor of the small-scale fading and the coefficients of its elevation law, Equation (6)
h(t), g(t), $J_{0}$	complex channel gain, unit-power Clarke (diffuse) process, Bessel function of the first kind and order zero
τ	age of a CQI report when it is used (1 to 7 ms by default)
$P_{\mathrm{ICI}}$	inter-carrier-interference power relative to the carrier, from the residual LoS Doppler shift
${\mathrm{SINR}}_{\mathrm{meas}}$, ${\mathrm{SINR}}_{\mathrm{fb}}$	SINR measured for a CQI report and the corrected value that is quantized (dB)
$\Delta (f_{D},\;K)$, α	calibrated CSI-aging back-off (dB) and its scale (1 unless stated)
$\delta_{u}$, $\Delta_{\mathrm{up}}$, *t*	OLLA offset of UE *u* (dB), its up-step (1 dB) and its BLER target (0.1)
Q(·), ${\mathrm{CQI}}_{\mathrm{eff}}$	Simu5G’s SINR-to-CQI quantizer and the CQI it reports

**Table 3:** Calibrated CSI-aging back-off $\Delta (f_{D},K)$ in dB for Simu5G’s default CQI window (report used 1 to 7 ms after it is taken), $10^{-2}$ BLER target and LTE-Sim TU BLER tables. Rows: Rician K-factor; columns: maximum Doppler frequency. The A2V module reads the row from the current LoS state and K(θ), the column from Equation (1).

$f_{D}$ (Hz)	0	10	25	50	100	150	200	300	400	500	600
NLoS (Rayleigh)	0.0	1.8	5.2	6.8	7.6	7.6	7.9	7.9	7.9	7.9	7.9
K=3 dB	0.0	0.9	3.8	5.8	6.0	6.2	6.3	6.5	6.6	6.6	6.6
K=6 dB	0.0	0.5	2.1	4.5	4.9	5.1	5.2	5.4	5.5	5.5	5.5
K=9 dB	0.0	0.2	1.0	2.3	2.8	3.0	3.1	3.3	3.5	3.5	3.5
K=12 dB	0.0	0.1	0.5	1.2	1.4	1.6	1.7	1.8	1.9	1.9	1.9
K=15 dB	0.0	0.1	0.2	0.6	0.7	0.8	0.9	0.9	1.0	1.0	1.0
K=20 dB	0.0	0.1	0.1	0.2	0.3	0.3	0.3	0.3	0.3	0.3	0.3

**Table 4:** Origin and experimental role of the software components used in the system-level evaluation.

Component	Source	Role in the Reported Evaluation
A2GChannel	new	air–ground physics of Section 4 (LoS sigmoid, path loss, shadowing, Rician process with dual-mobility Doppler, ICI); exercised on every aerial-DU link
A2VChannel	new	A2GChannel plus the Doppler-aware CQI correction of Equation (8), the OLLA comparison arm and the observables; installed on aerial DUs, terrestrial gNBs (V2I branch) and vehicles (cell selection); exercised on aerial and terrestrial links as specified in Section 4.
V2I branch	Simu5G TR 38.901 + new fading	path loss/LoS/shadowing reused; fading process and optional back-off new; exercised on every terrestrial link
V2VChannel	new	TR 37.885 sidelink path loss; compiled, not exercised (no sidelink traffic)
AerialDU node	new	Simu5G gNodeB structure with UAV3DMobility, small-cell power, E2 gate; exercised
UAV3DMobility	new	circular-patrol and waypoint (corridor) trajectories, altitude and energy model, flight envelope recorded; exercised
NearRTRIC	new	measurement plane (signal subscription, KPI aggregation, per-class split, latency histograms); xApps in shadow mode
NonRTRIC, rApps	new	A1 exchange, shadow mode; no effect on results
Aging calibration	new (offline)	Python 3.13.3 link-level study producing Table 3
NR UE, gNB stack, AMC, schedulers (PF, DRR, MAXCI), HARQ, RLC/PDCP, handover, X2, UPF	Simu5G	reused unchanged, except one robustness patch to the DRR scheduler (Appendix B)
Veins/SUMO coupling, INET IPv4 (GlobalArp)	Veins, INET	reused unchanged

**Table 5:** Modeling assumptions: what the experiments keep, simplify or omit, and why.

Aspect	Treatment	Reason
Vehicle mobility	kept	SUMO car-following on the road network; drives $f_{D}$, θ and handovers
UAV mobility	kept, simplified	3D patrol at constant altitude (circles in the grid, road legs on the highway); the energy model runs but no return-to-base occurs within 300 s
Large-scale channel	kept	Section 4 for A2V, TR 38.901 for V2I
Small-scale fading and CSI aging	kept	the object of study; identical on and off
CQI reporting	kept (Simu5G default)	6 TTI period, 1 TTI delay; varied in the sensitivity study
Uplink traffic	simplified	one CBR flow per vehicle (1500/2000 B every 10 ms); one slice populated
Downlink traffic	omitted	evaluation is restricted to uplink CQI aging and HARQ.
Inter-cell interference	omitted (Simu5G default)	isolates CSI-aging effects from interference-induced SINR variation; interference-aware evaluation is outside the present scope
Backhaul of aerial DUs	simplified	ideal 1 Gb/s link with 2 ms delay; the radio path is unaffected
E2/A1 encoding	simplified	signal subscription and typed messages instead of E2AP/ASN.1
xApp control	omitted	shadow mode; no claim about closed-loop control
Antenna patterns	simplified	omnidirectional 3 dBi (aerial), Simu5G default 18 dBi (terrestrial)
Handover algorithm	kept (Simu5G default)	RSSI-based with hysteresis; X2 full mesh added

**Table 6:** Network and physical-layer parameters shared by both scenarios.

Parameter	Value	Source
Carrier frequency $f_{c}$	3.5 GHz	3GPP TS 38.101-1, band n78
Numerology, subcarrier spacing	μ=1, 30 kHz	3GPP TS 38.211
Slot duration	0.5 ms	3GPP TS 38.211
CQI period/delay	6/1 TTI (Simu5G TTI = 1 ms)	Simu5G default
CQI target BLER	$10^{-2}$	Simu5G default
HARQ retransmission reduction	0.2 per retransmission	Simu5G default
gNB/aerial DU/UE transmit power	46/30/26 dBm	TR 38.901; small cell; TS 38.101
gNB/aerial DU antenna gain	18/3 dBi	Simu5G default; UAV patch
Noise figure UE/BS	7/5 dB	Simu5G default
Vehicle antenna height	1.5 m	TR 38.901 Table 7.4.1-1
Handover	RSSI-based, 50 ms latency, X2 mesh	Simu5G default
Near-RT measurement tick/A1 period	10 ms/1 s	O-RAN WG3/WG2
Warm-up/run length/seeds	10 s/300 s/5	this study

**Table 7:** Channel-model parameters and their sources.

Parameter	Value	Source
Sigmoid (a, b) urban/suburban	(9.61, 0.16)/(4.88, 0.43)	Al-Hourani et al. [10], Table I
Excess loss $\eta_{\mathrm{LoS}}$/$\eta_{\mathrm{NLoS}}$	1.0/20.0 dB	Al-Hourani et al. [10]
Shadowing $\sigma_{\mathrm{LoS}}$/$\sigma_{\mathrm{NLoS}}$	4.2/6.0 dB	Matolak and Sun [12]
LoS and shadowing correlation distance	50 m	Simu5G default
Rician K-factor coefficients $K_{1}$, $K_{2}$	2.0, 0.05 $\deg^{-1}$	fit to Matolak and Sun [12,13]
Fading paths, RMS delay spread	8, 50 ns	Clarke [18]; A2G delay spreads [12]
V2I model/LoS *K*	TR 38.901 UMa, RMa/9, 7 dB	3GPP TR 38.901 Tables 7.4.1-1, 7.5-6 [8]
V2V model (not exercised)	TR 37.885 highway/urban	3GPP TR 37.885
Back-off table $\Delta (f_{D},\;K)$	Table 3	link-level calibration (Section 4.5)
OLLA arm: $\Delta_{\mathrm{up}}$, *t*, offset range	1 dB, 0.1, [−3, 12] dB	conventional OLLA [21]; $t=10^{-2}$ variant in Section 7.7

**Table 8:** Per-scenario configuration and node counts. Vehicle counts are read from the recorded runs: the number of vehicles that entered during the 300 s (identical across seeds, because the SUMO demand definition is shared) and the mean number simultaneously present with the mean vehicle lifetime, obtained from each vehicle’s transmission time.

Parameter	Urban	Highway
Road network	1000×1000 m grid	3000 m straight road, 2×3 lanes
Terrestrial gNBs (count, mast height)	4 (2×2 pattern), 25 m	6 (500 m spacing, 35 m off the road), 30 m
V2I scenario	TR 38.901 UMa	TR 38.901 RMa
Aerial DUs	3	5
Cruise altitude/patrol speed/pattern	100 m/7.5 m/s/circle of 150 m radius	120 m/15 m/s/500 m back-and-forth road leg
LoS parameter set	urban	suburban
Vehicle speed range	20 to 60 km/h	60 to 140 km/h
PRBs per cell (30 kHz)	25	50
Vehicle CBR payload/period	1500 B/10 ms	2000 B/10 ms
CBR start time	uniform(5, 12) s	uniform(5, 12) s
Vehicles entering per run (identical across seeds)	244	93
Vehicles simultaneously present (mean; mean lifetime)	56 (66 s)	25 (77 s)

**Table 9:** Factorial results: mean ± 95% confidence-interval half-width over five seeds. HARQ = first-transmission error rate of uplink MAC PDUs (all UEs, aerial-served UEs and terrestrial-served UEs); delay = mean uplink MAC delay; first = share of PDUs delivered on the first transmission; throughput = per-UE MAC throughput (defined in Appendix A); PRB = carrier utilization. Feedback settings are off, back-off on A2V links only, back-off on all links, and OLLA on all links (comparison arm).

Scen.	Sched.	Feedback	HARQ (%)	HARQ Aer. (%)	HARQ Terr. (%)	Delay (ms)	First (%)	Thr. (kb/s)	PRB (%)
Urban	PF	off	18.4 ± 0.7	9.9 ± 0.6	20.9 ± 0.9	2.62 ± 0.03	78.2 ± 1.0	36.8 ± 6.6	36.9 ± 1.7
		A2V only	17.5 ± 0.4	5.1 ± 0.3	21.2 ± 0.5	2.58 ± 0.02	79.4 ± 0.7	37.0 ± 5.8	40.2 ± 1.4
		all links	7.9 ± 0.2	5.3 ± 0.4	8.7 ± 0.3	2.26 ± 0.01	89.2 ± 0.3	35.6 ± 5.8	45.3 ± 1.0
		OLLA	8.1 ± 0.4	6.2 ± 1.0	8.7 ± 0.3	2.27 ± 0.02	89.2 ± 0.6	34.5 ± 4.7	48.0 ± 2.0
	DRR	off	18.1 ± 0.7	10.4 ± 1.3	20.3 ± 0.8	2.60 ± 0.02	79.2 ± 0.7	37.3 ± 5.4	36.6 ± 1.7
		A2V only	17.1 ± 0.5	5.7 ± 0.6	20.5 ± 0.4	2.57 ± 0.02	80.4 ± 0.6	36.4 ± 7.8	40.2 ± 1.6
		all links	6.9 ± 0.3	5.0 ± 0.5	7.5 ± 0.3	2.22 ± 0.01	90.6 ± 0.3	35.9 ± 6.9	45.5 ± 1.9
		OLLA	7.9 ± 0.5	6.0 ± 0.7	8.5 ± 0.6	2.26 ± 0.02	89.7 ± 0.5	36.1 ± 7.4	49.3 ± 2.7
	MAXCI	off	18.5 ± 1.1	10.7 ± 1.9	20.9 ± 1.2	2.62 ± 0.04	77.9 ± 1.5	37.4 ± 5.1	36.7 ± 1.8
		A2V only	17.4 ± 0.2	4.9 ± 0.5	21.0 ± 0.2	2.58 ± 0.01	78.9 ± 0.1	36.2 ± 8.0	39.8 ± 0.3
		all links	7.3 ± 0.2	5.5 ± 0.5	7.9 ± 0.2	2.24 ± 0.01	89.4 ± 0.4	35.2 ± 7.1	43.9 ± 1.5
		OLLA	7.6 ± 0.2	6.2 ± 0.8	8.1 ± 0.2	2.25 ± 0.01	89.5 ± 0.4	36.1 ± 7.8	47.5 ± 1.6
Highway	PF	off	2.0 ± 0.4	0.8 ± 0.2	2.7 ± 0.6	2.07 ± 0.01	97.7 ± 0.5	38.5 ± 20.0	4.1 ± 0.4
		A2V only	1.7 ± 0.5	0.3 ± 0.0	2.7 ± 0.8	2.06 ± 0.02	97.8 ± 0.7	38.5 ± 20.3	4.2 ± 0.3
		all links	0.9 ± 0.2	0.2 ± 0.1	1.4 ± 0.4	2.05 ± 0.02	98.3 ± 0.5	38.6 ± 20.7	4.3 ± 0.3
		OLLA	2.1 ± 0.1	2.2 ± 0.0	1.9 ± 0.3	2.09 ± 0.01	97.1 ± 0.3	37.1 ± 20.0	4.1 ± 0.1
	DRR	off	2.1 ± 0.3	0.8 ± 0.2	2.9 ± 0.5	2.07 ± 0.01	97.6 ± 0.7	38.8 ± 20.5	4.1 ± 0.3
		A2V only	1.9 ± 0.2	0.2 ± 0.0	3.2 ± 0.4	2.06 ± 0.01	97.8 ± 0.5	37.8 ± 20.1	4.1 ± 0.1
		all links	1.0 ± 0.3	0.3 ± 0.1	1.4 ± 0.5	2.04 ± 0.02	98.3 ± 0.5	37.9 ± 19.4	4.2 ± 0.3
		OLLA	2.3 ± 0.2	2.2 ± 0.1	2.2 ± 0.4	2.09 ± 0.01	96.8 ± 0.6	37.5 ± 19.5	4.3 ± 0.3
	MAXCI	off	2.0 ± 0.3	0.8 ± 0.1	2.8 ± 0.5	2.07 ± 0.01	97.7 ± 0.3	37.2 ± 19.7	4.0 ± 0.2
		A2V only	1.6 ± 0.3	0.2 ± 0.1	2.7 ± 0.4	2.06 ± 0.01	98.1 ± 0.5	37.9 ± 20.1	4.1 ± 0.2
		all links	0.8 ± 0.2	0.2 ± 0.0	1.3 ± 0.4	2.04 ± 0.02	98.7 ± 0.4	38.6 ± 19.8	4.1 ± 0.2
		OLLA	2.2 ± 0.1	2.2 ± 0.1	2.1 ± 0.2	2.10 ± 0.02	97.1 ± 0.3	37.6 ± 19.7	4.1 ± 0.1

**Table 10:** Paired differences matched by scheduler and seed: mean ± 95% confidence-interval half-width over five seed pairs. The first three rows of each scheduler are feedback setting minus off; the row labeled “all links vs OLLA” is the back-off on all links minus OLLA, the direct comparison of the two corrections. The symbols *, ** and *** denote p<0.05, p<0.01 and p<0.001, respectively, in paired *t*-tests. Negative values indicate reductions and positive values indicate increases. Units are as in Table 9: percentage points for rates, ms for delay and kb/s for throughput.

Scen.	Sched.	Feedback	ΔHARQ	ΔHARQ Aer.	ΔHARQ Terr.	ΔDelay	ΔFirst	ΔThr.	ΔPRB
Urban	PF	A2V only	−1.0 ± 0.9 *	−4.8 ± 0.7 ***	+0.2 ± 1.0	−0.04 ± 0.03 *	+1.2 ± 1.2	+0.2 ± 1.8	+3.3 ± 2.0 *
	PF	all links	−10.6 ± 0.8 ***	−4.5 ± 0.7 ***	−12.3 ± 0.9 ***	−0.35 ± 0.03 ***	+11.0 ± 1.0 ***	−1.2 ± 1.5	+8.4 ± 1.5 ***
	PF	OLLA	−10.3 ± 0.7 ***	−3.7 ± 1.4 **	−12.2 ± 0.8 ***	−0.35 ± 0.03 ***	+11.0 ± 1.0 ***	−2.3 ± 2.4	+11.1 ± 2.1 ***
	PF	all links vs OLLA	−0.2 ± 0.5	−0.9 ± 1.2	−0.0 ± 0.5	−0.01 ± 0.02	−0.0 ± 0.8	+1.1 ± 2.1	−2.7 ± 2.5 *
	DRR	A2V only	−1.0 ± 0.5 **	−4.7 ± 1.0 ***	+0.1 ± 0.8	−0.03 ± 0.02 *	+1.2 ± 0.8 *	−0.9 ± 3.6	+3.6 ± 1.7 **
	DRR	all links	−11.2 ± 0.6 ***	−5.4 ± 1.3 ***	−12.8 ± 0.6 ***	−0.38 ± 0.02 ***	+11.5 ± 0.7 ***	−1.4 ± 4.3	+8.8 ± 2.2 ***
	DRR	OLLA	−10.2 ± 0.4 ***	−4.4 ± 0.9 ***	−11.8 ± 0.4 ***	−0.34 ± 0.02 ***	+10.5 ± 0.4 ***	−1.2 ± 5.0	+12.6 ± 1.6 ***
	DRR	all links vs OLLA	−1.0 ± 0.3 ***	−1.0 ± 0.8 *	−1.0 ± 0.4 **	−0.03 ± 0.01 **	+1.0 ± 0.3 **	−0.2 ± 1.4	−3.8 ± 1.7 **
	MAXCI	A2V only	−1.1 ± 1.0 *	−5.8 ± 1.8 ***	+0.1 ± 1.3	−0.04 ± 0.04 *	+1.0 ± 1.5	−1.2 ± 4.3	+3.1 ± 1.8 **
	MAXCI	all links	−11.2 ± 1.0 ***	−5.2 ± 2.3 **	−13.0 ± 1.0 ***	−0.38 ± 0.04 ***	+11.5 ± 1.4 ***	−2.2 ± 4.0	+7.3 ± 2.4 **
	MAXCI	OLLA	−10.9 ± 1.1 ***	−4.5 ± 1.3 ***	−12.8 ± 1.2 ***	−0.36 ± 0.04 ***	+11.6 ± 1.7 ***	−1.3 ± 4.8	+10.8 ± 2.9 ***
	MAXCI	all links vs OLLA	−0.3 ± 0.3	−0.7 ± 1.1	−0.2 ± 0.3	−0.01 ± 0.01	−0.1 ± 0.8	−0.9 ± 1.1	−3.6 ± 2.2 *
Highway	PF	A2V only	−0.3 ± 0.2 *	−0.5 ± 0.2 ***	+0.0 ± 0.5	−0.01 ± 0.01	+0.1 ± 0.4	−0.1 ± 1.6	+0.1 ± 0.3
	PF	all links	−1.1 ± 0.5 **	−0.6 ± 0.2 **	−1.3 ± 0.8 **	−0.02 ± 0.02	+0.7 ± 0.8	+0.0 ± 1.1	+0.2 ± 0.5
	PF	OLLA	+0.1 ± 0.4	+1.3 ± 0.2 ***	−0.8 ± 0.7 *	+0.03 ± 0.02 *	−0.6 ± 0.7	−1.4 ± 1.8	+0.1 ± 0.5
	PF	all links vs OLLA	−1.2 ± 0.1 ***	−1.9 ± 0.0 ***	−0.5 ± 0.2 ***	−0.05 ± 0.02 **	+1.3 ± 0.3 ***	+1.4 ± 1.7	+0.1 ± 0.2
	DRR	A2V only	−0.2 ± 0.3	−0.6 ± 0.2 **	+0.2 ± 0.3	−0.01 ± 0.01	+0.2 ± 0.6	−1.0 ± 1.5	+0.0 ± 0.3
	DRR	all links	−1.2 ± 0.5 **	−0.6 ± 0.3 **	−1.5 ± 0.9 *	−0.03 ± 0.03	+0.8 ± 1.2	−0.9 ± 3.2	+0.1 ± 0.6
	DRR	OLLA	+0.1 ± 0.4	+1.4 ± 0.2 ***	−0.7 ± 0.7 *	+0.02 ± 0.02 *	−0.8 ± 1.0	−1.3 ± 3.2	+0.2 ± 0.5
	DRR	all links vs OLLA	−1.3 ± 0.3 ***	−1.9 ± 0.1 ***	−0.8 ± 0.5 **	−0.05 ± 0.01 ***	+1.6 ± 0.6 **	+0.4 ± 1.4	−0.1 ± 0.3
	MAXCI	A2V only	−0.3 ± 0.4	−0.6 ± 0.2 ***	−0.1 ± 0.6	−0.01 ± 0.02	+0.4 ± 0.7	+0.7 ± 1.3	+0.1 ± 0.3
	MAXCI	all links	−1.1 ± 0.3 ***	−0.6 ± 0.1 ***	−1.5 ± 0.6 **	−0.03 ± 0.01 **	+1.0 ± 0.4 **	+1.4 ± 1.4 *	+0.0 ± 0.2
	MAXCI	OLLA	+0.2 ± 0.3	+1.4 ± 0.1 ***	−0.7 ± 0.5 *	+0.03 ± 0.02 *	−0.6 ± 0.5 *	+0.4 ± 2.8	+0.1 ± 0.2
	MAXCI	all links vs OLLA	−1.3 ± 0.3 ***	−2.0 ± 0.1 ***	−0.8 ± 0.5 **	−0.06 ± 0.03 **	+1.6 ± 0.6 **	+1.0 ± 1.6	−0.1 ± 0.3

**Table 11:** Two-way ANOVA results by scenario and KPI: *F* statistic (*p*-value) for the scheduler main effect (2 degrees of freedom), feedback main effect (3 degrees of freedom) and scheduler-by-feedback interaction (6 degrees of freedom), with 48 residual degrees of freedom.

Scenario	KPI	Scheduler	Feedback	Interaction
Urban	HARQ error rate	6.4 (0.003)	3019.9 (<0.001)	1.6 (0.156)
Urban	MAC delay	6.7 (0.003)	2229.6 (<0.001)	1.2 (0.336)
Urban	First-transmission share	22.9 (<0.001)	1859.5 (<0.001)	1.8 (0.114)
Urban	Per-UE throughput	0.0 (0.964)	0.3 (0.808)	0.1 (0.999)
Urban	PRB utilization	2.4 (0.105)	208.9 (<0.001)	0.7 (0.683)
Highway	HARQ error rate	2.6 (0.086)	98.8 (<0.001)	0.3 (0.938)
Highway	MAC delay	0.6 (0.563)	55.9 (<0.001)	0.5 (0.794)
Highway	First-transmission share	2.0 (0.153)	35.5 (<0.001)	0.3 (0.915)
Highway	Per-UE throughput	0.0 (0.998)	0.0 (0.999)	0.0 (1.000)
Highway	PRB utilization	2.2 (0.122)	1.4 (0.244)	0.5 (0.828)

**Table 12:** Operating-regime summary of the recorded A2V observables. Values are the medians, with the 10th- to 90th-percentile ranges in parentheses, of the pooled distributions shown in Figure 9.

Observable	Urban	Highway
Maximum Doppler $f_{D}$ (Hz)	165.0 (63.0 to 243.0)	232.8 (148.3 to 524.6)
LoS Doppler shift $f_{d}$ (Hz)	−22.0 (−142.0 to 86.0)	−120.0 (−400.0 to 110.0)
Elevation angle (°)	38.1 (18.7 to 72.3)	52.0 (27.1 to 80.8)
Rician *K*-factor, LoS links (dB)	12.0 (8.0 to 18.0)	14.3 (8.9 to 20.4)
Computed back-off Δ (dB)	1.3 (0.3 to 7.5)	1.0 (0.2 to 3.3)
Relative speed (m/s)	14.3 (5.5 to 20.7)	20.0 (12.4 to 44.9)

## Data Availability

The OMNeT++/Simu5G/Veins source code of the framework, the link-level calibration script, the per-run scalar recordings of every experiment reported here, and the scripts that produced every table and figure are available from the corresponding author upon reasonable request. They will be deposited in a public repository.

## Abbreviations

The following abbreviations are used in this manuscript:

3GPP	3rd Generation Partnership Project
A2G	air-to-ground
A2V	air-to-vehicle
ACK/NACK	acknowledgment/negative acknowledgment
AMC	adaptive modulation and coding
ANOVA	analysis of variance
ASN.1	Abstract Syntax Notation One
BLER	block error rate
CBR	constant bit rate
CDF	cumulative distribution function
CI	confidence interval
CQI	channel quality indicator
CSI	Channel State Information
DRR	deficit round robin
DU	distributed unit
E2AP	E2 Application Protocol
eMBB	enhanced mobile broadband
FSPL	free-space path loss
gNB	next-generation NodeB
HARQ	Hybrid Automatic Repeat Request
ICI	inter-carrier interference
IP	Internet Protocol
ITS	intelligent transportation systems
KPI	key performance indicator
LiDAR	light detection and ranging
LoS/NLoS	line of sight/non-line of sight
LTE	Long-Term Evolution
MAC	Medium Access Control
MAXCI	maximum carrier-to-interference scheduler
MCS	modulation and coding scheme
NR	New Radio
OFDMA	orthogonal frequency-division multiple access
OLLA	outer-loop link adaptation
O-RAN	Open Radio Access Network
PDCP	packet data convergence protocol
PDU	protocol data unit
PF	proportional fair
PHY	physical layer
PRB	physical resource block
RAN	radio access network
RB	resource block
rApp	Non-RT RIC application
RIC	RAN Intelligent Controller (Non-RT: non-real-time; Near-RT: near-real-time)
RLC	radio link control
RMa/UMa	rural macro/urban macro
RMS	root mean square
RSSI	received signal strength indicator
RSU	roadside unit
SINR	signal-to-interference-plus-noise ratio
SUMO	Simulation of Urban MObility
TR	Technical Report
TS	Technical Specification
TTI	transmission time interval
TTT	time-to-trigger
TU	typical urban
UAV	unmanned aerial vehicle
UE	user equipment
UPF	user plane function
V2I/V2V/V2X	vehicle-to-infrastructure/vehicle-to-vehicle/vehicle-to-everything
xApp	Near-RT RIC application

## Appendix A. Measurement-Plane Implementation and KPI Definitions

This appendix defines the measurement-plane processing used for the KPIs in Section 7. The Near-RT RIC implements the OMNeT++ cListener interface and subscribes at the network module to the Simu5G signals harqErrorRate_1st_Ul, macDelayUl, macThroughputUl, avgServedBlocksUl and servingCell, as well as to the A2V channel observables. Because OMNeT++ signals propagate upward through the module hierarchy, one network-level subscription captures every vehicle spawned during a run. The recorded observables retain their implementation identifiers; for example, a2vAgingMarkdownDb is the computed candidate back-off Δ, which is subtracted before CQI quantization only when the Doppler-aware feedback is enabled. Under the OLLA arm, the per-UE offset (a2vOllaOffsetDb, v2iOllaOffsetDb) takes its place. At every 10 ms tick, the RIC aggregates per-UE and per-cell measurements, while servingCell supports aerial/terrestrial classification and handover counting.

Uplink MAC-delay samples are accumulated in fixed 0.1 ms bins, and percentiles are read from the corresponding upper bin edges. PRB utilization is the number of resource blocks served per TTI divided by the carrier size. For every correctly received uplink MAC PDU, Simu5G emits the UE’s cumulative received bytes divided by the time elapsed since the end of the warm-up period. At each tick, the RIC averages the samples of each UE and then calculates an equal-weight mean over the active UEs; the run-level throughput is the time average of these tick-level values. Because the denominator begins at the end of the warm-up period rather than at each vehicle’s entry time, late-entering vehicles contribute lower values. This entry-time effect is common to matched treatments but increases the seed-to-seed spread when few vehicles are present simultaneously.

## Appendix B. Reproducibility and Compatibility Notes

The project uses an OMNeT++ multi-project layout with sibling checkouts of OMNeT++, INET, Veins and Simu5G. The source code, release-build configuration, scenario files, batch scripts, calibration script and recorded results will be made available together. Each simulation run is identified by its configuration name, run number and seed set. The SUMO seed equals the repetition index so that matched treatments use the same traffic realization.

Two software compatibility measures are required. First, the evaluated software stack uses INET’s GlobalArp because the default address resolution protocol (ARP) module passes packets to Simu5G’s IP-to-network-interface module that it cannot parse in the larger topology. GlobalArp resolves addresses through a global table without modifying the radio path. Second, Simu5G’s deficit-round-robin scheduler retains stale connections after a UE hands over, causing an exception in the evaluated topology. A nine-line patch removes these connections from the scheduler’s active list and will be included with the source code. These measures preserve the channel models, feedback correction and KPI definitions while ensuring valid address resolution and scheduler-state management.
